# Retinoic acid and RARγ maintain satellite cell quiescence through regulation of translation initiation

**DOI:** 10.1038/s41419-022-05284-9

**Published:** 2022-09-29

**Authors:** Wenzhe Luo, Yueyuan Xu, Ruige Liu, Yinlong Liao, Sheng Wang, Haoyuan Zhang, Xinyun Li, Heng Wang

**Affiliations:** 1 Key Laboratory of Agricultural Animal Genetics, Breeding, and Reproduction of the Ministry of Education, College of Animal Science and Technology, Huazhong Agricultural University, Wuhan, China; 2 College of Animal Science and Technology, Shandong Agricultural University, Taian, China

**Keywords:** Muscle stem cells, Quiescence

## Abstract

In adult skeletal muscle, satellite cells are in a quiescent state, which is essential for the future activation of muscle homeostasis and regeneration. Multiple studies have investigated satellite cell proliferation and differentiation, but the molecular mechanisms that safeguard the quiescence of satellite cells remain largely unknown. In this study, we purposely activated dormant satellite cells by using various stimuli and captured the in vivo-preserved features from quiescence to activation transitions. We found that retinoic acid signaling was required for quiescence maintenance. Mechanistically, retinoic acid receptor gamma (RARγ) binds to and stimulates genes responsible for Akt dephosphorylation and subsequently inhibits overall protein translation initiation in satellite cells. Furthermore, the alleviation of retinoic acid signaling released the satellite cells from quiescence, but this restraint was lost in aged cells. Retinoic acid also preserves the quiescent state during satellite cell isolation, overcoming the cellular stress caused by the isolation process. We conclude that active retinoic acid signaling contributes to the maintenance of the quiescent state of satellite cells through regulation of the protein translation initiation process.

## Introduction

Skeletal muscle contains a population of tissue-resident adult stem cells (also called satellite cells or muscle stem cells, MuSCs), which are required for the hypertrophy, metabolism and regeneration of skeletal muscle. Under homeostasis, MuSCs are normally maintained in a quiescent state. Upon injury, MuSCs can swiftly escape from dormancy to proliferate and differentiate, which generate numerous progenies to fuse with neighboring myofibers to repair damaged muscle fibers [1]. On the other hand, the MuSC pool must be maintained to endure repeated injuries, and this is achieved by replenishment with a population of daughter cells produced by the asymmetric division of activated MuSCs [2]. Importantly, quiescence is not simply an inactive state but a homeostatic state dynamically regulated by a combination of intrinsic and extrinsic factors. Quiescent MuSCs are distinguished by their specific high expression of Pax7 at both the RNA and protein levels [3, 4], while the canonical myogenic activator MyoD and Myf5 are repressed [5]. At the chromatin level, nearly 50% of annotated genes are marked by active chromatin histone (H3K4me3) modifications, which indicate that the chromatin state of quiescent MuSCs is relatively permissive in general [6]. Several reports have suggested that Notch signaling regulates quiescence by upregulating Pax7 and suppressing MyoD through action of the Notch intracellular domain (NICD) [7].

Recent studies showed that MuSCs experienced cellular stress [8, 9] and altered their transcriptome [9, 10] and proteome [11] patterns within hours during isolation, thus preventing us from discovering bona fide quiescence maintenance-associated factors in vivo. Moreover, the microenvironment niche that facilitates the in vivo quiescent-to-activation switch could not be preserved in the isolated cells. Therefore, strict preservation of the quiescence state during MuSC isolation is preferred, and in situ fixation before isolation was practiced in this study.

Vitamin A (retinol) and its derivative retinoic acid (RA), which was found over 100 years ago [12], are currently receiving considerable interest because of their important role in hematopoietic stem cell dormancy entry and exit [13], visual system function [14], cellular growth and development [15, 16], immune function [17, 18], and reproduction [19, 20]. Vitamin A/retinol is converted to RA by two sequential oxidation steps and then transported to the nucleus, where it binds to the retinoic acid receptor (RAR), which belongs to the superfamily of ligand-inducible transcriptional regulators [21, 22]. The RAR binds to specific DNA sequences of retinoic acid-responsive genes, called retinoic acid response elements (RAREs), and interacts coordinately with a large number of cofactors and corepressors to regulate target gene expression [23, 24]. The retinol and RARs have been extensively studied in various adult stem cells, including hematopoietic stem cells [25], breast cancer cells [26] and hepatocellular carcinoma cells [27], but the specific role of retinoic acid signaling in MuSCs remains controversial since both enhanced stemness and differentiation by RA have been reported [28, 29].

In this study, in situ transcriptome analysis of quiescent and activated MuSCs revealed that retinoic acid signaling is essential for quiescent state maintenance. Moreover, RA and RAR overexpression attenuate MuSC proliferation and differentiation, which is caused by reduced cellular protein synthesis through the RARγ/Akt/eIF4EBP1 signaling cascade. We further showed that the blockade of retinoic acid signaling in quiescent MuSCs in vivo causes them to re-enter the cell cycle. Altogether, these findings provide mechanistic insights that RA and RARγ are essential for the regulation of MuSC quiescence maintenance.

## Results

### Retinol metabolism is enriched in quiescent satellite cells

**Fig. 1:**
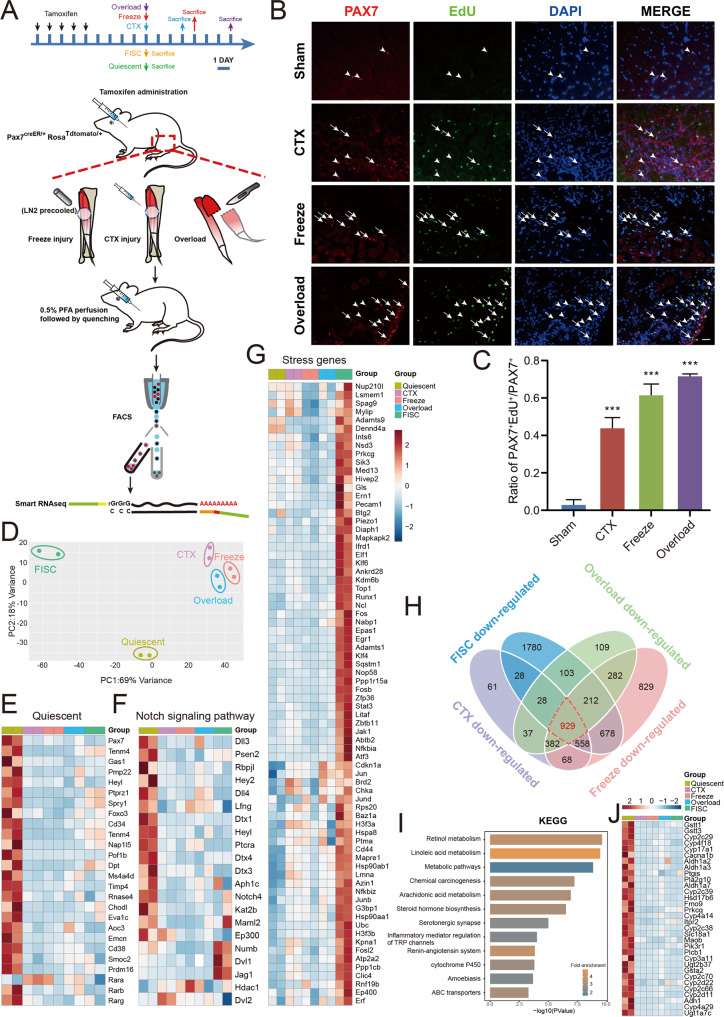
Retinol metabolism is enriched in quiescent satellite cells. **A** Experimental study design. Eight to 12-week-old Pax7^creER/+^; Rosa26 ^tdTomato/+^ mice were administrated with tamoxifen for five consecutive days followed by 5-day waiting period to label MuSCs with tdTomato. Quiescent and freshly isolated MuSCs were collected immediately after 5-day waiting period. MuSCs from CTX injury-, freeze injury- and overload-mouse models were harvested 3-, 4- and 7-day after surgery, respectively. Quiescent MuSCs as well as MuSCs from CTX injury-, freeze injury- and overload-mouse model representing cells in vivo state were fixed by PFA perfusion before isolation process. Freshly isolated MuSCs were collected without fixation. RNA-seq library of MuSCs sorted by FACS were constructed by Smart-RNAseq. CTX, cardiotoxin; PFA, paraformaldehyde; FACS, fluorescence activated cell sorting. **B**, **C** Representative pictures showing EdU and PAX7 staining in the sham model and different injury models. Arrows represent EdU^+^/PAX7^+^ cells; arrowheads represent EdU^-^/PAX7^+^ cells. **C** Quantification of EdU-positive MuSCs. *n* = 3 mice/treatment, >450 Pax7^+^ cells counted/mouse. **D** Principal component analysis (PCA) of the gene expression in quiescent MuSCs, CTX-injury MuSCs, freeze-injury MuSCs, overloaded MuSCs and FISCs. FISCs, freshly isolated satellite cells. *n* = 2 biological replicate RNA-seq data/group. **E**–**G** Expression heatmap of (**E**) quiescence-, (**F**) Notch signaling pathway- and (**G**) stress-associated genes induced by the isolation process. *n* = 2 biological replicate RNA-seq data/group. **H** Venn diagram of differentially downregulated genes in different activated MuSCs compared to quiescent cells. The red dashed area represents quiescence-maintaining genes. **I** Kyoto Encyclopedia of Genes and Genomes pathway analysis of quiescence-maintaining genes in **H**. The top 12 categories are shown. **J** Heatmap of representative gene expression for the retinol metabolism signaling pathway. *n* = 2 biological replicate RNA-seq data/group. Scale bar: 50 µm in **B**. Statistical results are expressed as the mean ± SEM. ***p < 0.001.

MuSCs rapidly escape quiescence and are activated during enzymatic dissociation [9, 10], thus preventing us from analyzing the native features of quiescent and activated MuSCs inside the muscles under physiological conditions. To preserve and acquire the MuSCs in a life-like state, we employed the light fixation (0.5% paraformaldehyde perfusion) protocol [5] in Pax7^creER/+^; Rosa26^tdTomato/+^ mouse, in which the majority of MuSCs were specifically marked with tdTomato after tamoxifen administration (Fig. S1A). Further, we used various tools to activate the MuSCs in vivo to explore their original and conserved quiescent identity in retrospect. Before muscle dissociation and FACS, MuSCs were activated by different types of insults, such as cardiotoxin (CTX) injury, freeze injury and mechanical overload (synergist ablation) (Fig. 1A). MuSCs from CTX injury-, freeze injury- and overload-mouse model were harvested 3-, 4- and 7-day after surgery, respectively. We also performed the EdU assay to confirm that the quiescent MuSCs were robustly activated in vivo in all injury models (Fig. 1B, C), although to varying degrees. In addition, viable freshly isolated satellite cells (FISCs) were collected after PBS perfusion to represent the in vitro-activated cells (Fig. S1B).

Next, the fixed quiescent MuSCs and different types of activated MuSCs were isolated by FACS and subjected to detailed gene expression analysis by Smart-seq. Principal component analysis (PCA) of the overall transcriptome showed remarkable separation between quiescent cells and activated cells. Interestingly, the transcriptomes of CTX-, freezing- and overload-induced cells were relatively similar, but the FISCs cells were clearly separated from the injury-activated MuSCs (Fig. 1D). This finding indicates that MuSCs are distinctively activated in vivo and in vitro.

To uncover the quiescent signatures of MuSCs in vivo, we comparatively analyzed the transcriptome data of quiescent cells and different types of activated cells. As expected, canonical quiescent marker genes, such as Pax7, Spry1, Tenm4 and CD34, were all expressed at the highest level in quiescent cells (Fig. 1E). Moreover, the Notch signaling genes that have been demonstrated to be associated with the quiescent state of various cells were also highly expressed in our quiescent MuSCs (Fig. 1F). In contrast, cell cycle-related genes, such as Mcm2, Cdk1 and Ccnb1, were expressed at low levels in quiescent cells (Fig. S1C). Importantly, stress response genes induced by the MuSC isolation process [8] were only highly expressed in FISCs but not in any of the in vivo-fixed cells, indicating that the light-fixation method unbiasedly preserved the native features of MuSCs (Fig. 1G).

To determine the key genes necessary for maintaining the genuine quiescent state of MuSCs, we tried to identify factors present only in quiescent cells but not in activated cells either in vivo or in vitro. Differential gene expression analysis of different types of activated and quiescent MuSCs revealed a total of 929 genes that were uniquely and highly expressed in quiescent cells (Fig. 1H). Kyoto Encyclopedia of Genes and Genomes (KEGG) pathway analysis further revealed that the most significantly enriched signaling pathway associated with the maintenance of the MuSC quiescent state was retinol metabolism (sequential oxidation of retinol to retinoic acid), whose genes (Adh1, Aldh1a2, Aldh1a3, Aldh1a7 and Hsd17b6) were specifically expressed in quiescent cells (Figs. 1I, J). In addition to retinol metabolism, polyunsaturated fatty acid metabolism (linoleic and arachidonic acid), steroid hormone synthesis pathways, and ATP-binding cassette (ABC) transporter signaling pathways were also enriched and highly expressed in quiescent MuSCs. Together, the analysis of transcriptomic data on quiescent and activated cells revealed a potential contribution of retinol and its oxidation product RA to quiescence maintenance in MuSCs.

### Retinoic acid and RARγ inhibit satellite cell proliferation and differentiation

**Fig. 2:**
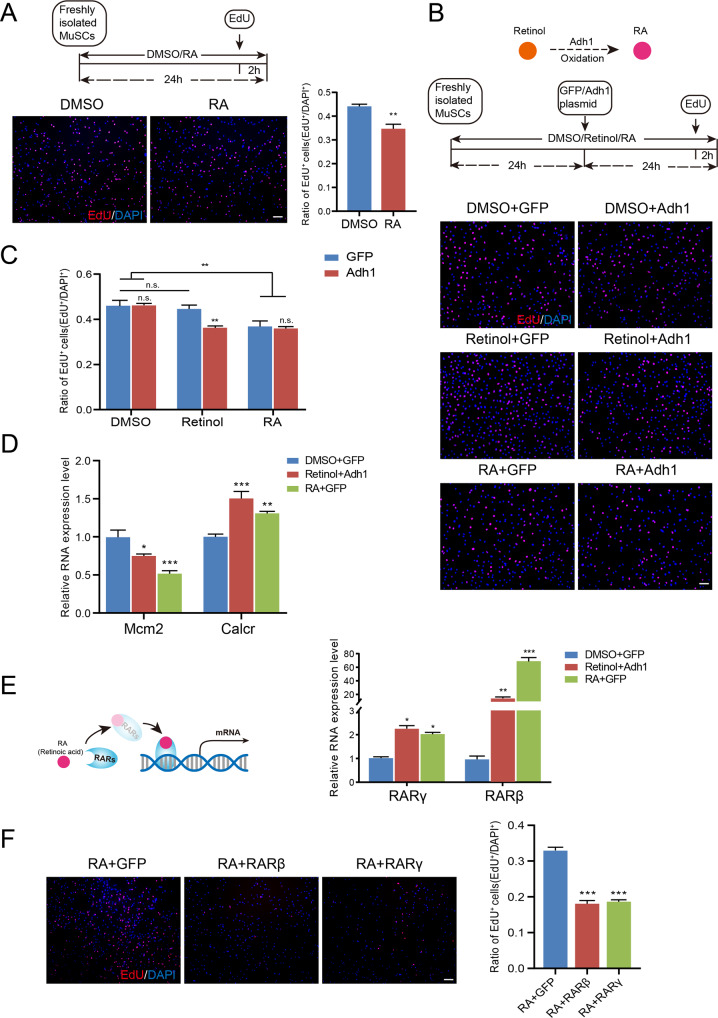
Retinoic acid and RARγ inhibit satellite cell proliferation and differentiation. **A** Upper: experimental study design. Bottom left: Representative pictures showing EdU assays in MuSCs treated with RA or DMSO. Bottom right: Quantification of EdU-positive MuSCs. *n* = 3 independent assays/condition, >3000 cells counted/assay. **B**, **C** Upper: the oxidation of retinol to RA by dehydrogenases, including ADH1. Middle: experimental study design. Bottom: representative pictures showing EdU staining after different treatments, including DMSO + GFP, DMSO + Adh1, Retinol+GFP, Retinol+Adh1, RA + GFP and RA + Adh1. **C** Quantification of EdU-positive MuSCs. *n* = 3 independent assays/condition, >3000 cells counted/assay. **D**, **E** RT–qPCR analysis of marker genes of the cell cycle (Mcm2) and quiescence (Calcr). **E** The mRNA expression of retinoic acid receptors (RARβ and RARγ) in DMSO/GFP-, Retinol/Adh1-, and RA/GFP-treated cells. The left panel shows that RA transcriptionally regulates gene expression through RARs. *n* = 3 independent assays. **F** Left: Representative pictures showing the EdU assay in RA-treated MuSCs overexpressing RARβ, RARγ or GFP. Right: Quantification of EdU-positive MuSCs. *n* = 3 independent assays/condition, >3000 cells counted/assay. Scale bar: 50 µm in **A** and **B**, 100 μm in **F**. Statistical results are expressed as the mean ± SEM. n.s., not significant. *p < 0.05, **p < 0.01, ***p < 0.001.

We examined the proliferation and differentiation potentials of MuSCs treated with RA or DMSO to determine whether retinol and its oxidation product RA contribute to the maintenance of the cell cycle-arrest state of quiescent MuSCs. EdU assay in vitro showed that the rate of proliferating MuSCs decreased from an average of 44% in the control group to 34% after RA treatment (Fig. 2A). Also, RA decreased the proportion of proliferating single fiber-associated MuSCs (Mcm2^+^/PAX7^+^) 24 h after muscle tissue was dissected (Fig. S2A). Consistently, cell cycle analysis based on DNA content also showed that RA treatment maintained more MuSCs in G0/G1 state rather than transitioning to S phase (Fig. S2B, C). The differentiation capacity of the MuSCs was also severely impaired upon RA treatment, as evidenced by a decreased fusion index calculated by counting the proportion of nuclei in myotubes with at least three nuclei (Fig. S2D, E). Therefore, forcibly enhancing RA signaling with the exogenous addition of RA could immobilize MuSCs and promote a quiescent state in vitro and ex vivo.

Since RA is derived from the sequential oxidation of retinol by specific dehydrogenases and the transcriptome data indicate that the enzymes that continuously oxidize retinol to RA are highly expressed in the quiescent state (Fig. 1J), we examined the expression of alcohol dehydrogenase (Adh1), a key rate-limiting enzyme that oxidizes retinol to retinaldehyde, in quiescent and in vitro-activated MuSCs using RT–qPCR, and the results showed that the expression level of Adh1 was significantly decreased in activated cells (Fig. S2F). Next, we investigated whether Adh1 is involved in quiescence maintenance by overexpressing Adh1 in MuSCs (Fig. S2G). We found that cell proliferation was significantly decreased when both retinol and Adh1 were administered to MuSCs, as shown by the EdU assay. In contrast, no significant change in MuSC proliferation was observed when Adh1 was overexpressed but without retinol, indicating that the initial substrate, retinol, was necessary for activating RA signaling to promote quiescence. On the other hand, the overexpression of Adh1 after RA treatment did not further reduce cell proliferation compared with RA alone, confirming that RA is the effector molecule produced by ADH1 to initiate downstream signaling for quiescence maintenance (Fig. 2B, C). In addition, the expression of the proliferation marker Mcm2 was significantly reduced in the retinol+Adh1- and RA-treated cells, whereas the expression of the quiescence marker Calcr was increased (Fig. 2D). These results suggest that Adh1 is necessary for the oxidation of retinol to RA for quiescence maintenance.

Previous studies have shown that RA acts as the ligand to form complexes with nuclear receptors to bind DNA elements to induce target gene expression [30]. Accordingly, we also found that the expression of retinoic acid receptors (RARβ and RARγ) was significantly higher in quiescent MuSCs than in other activated cells in vivo (Fig. 1E). In addition, MuSCs treated with retinol+Adh1 or RA expressed higher levels of both receptors than control cells (Fig. 2E). Correspondingly, the overexpression of RARβ or RARγ in the presence of RA significantly inhibited both cell proliferation (Fig. 2F) and differentiation (Fig. S2J) compared to RA treatment alone. The overexpression efficiency of both receptors was validated using RT-qPCR (Fig. S2H, I). Collectively, these results suggest that RA signaling via the actions of RARs prevents cell proliferation and differentiation, shifting MuSCs to a quiescent-like state.

### Retinoic acid and RARγ inhibit MyoD protein synthesis

**Fig. 3:**
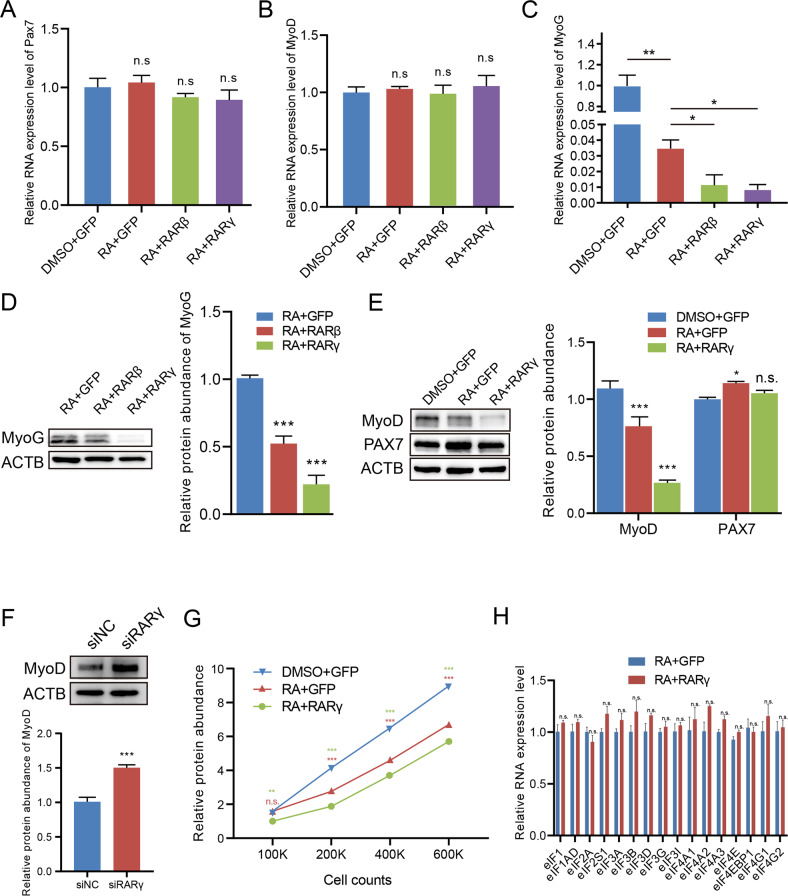
Retinoic acid and RARγ inhibit MyoD protein synthesis. **A**–**C** RT–qPCR analysis of (**A**) Pax7, (**B**) MyoD and (**C**) MyoG in DMSO-treated cells overexpressing GFP and RA-treated cells overexpressing GFP, RARβ or RARγ. n = 3 independent assays. **D** Left: western blot analysis of MyoG in RA-treated MuSCs overexpressing GFP, RARβ or RARγ. ACTB (β-actinin) was used as the control. Right: Integrated density of Western blot bands was quantified. *n* = 3 independent assays. **E** Left: western blot analysis of MyoD and PAX7 in DMSO + GFP-, RA + GFP-, RA + RARβ- or RA + RARγ-treated MuSCs. Right: Integrated density of Western blot bands was quantified. *n* = 3 independent assays. **F** Upper: western blot analysis of MyoD in MuSCs transfected with siRARγ or siNC. Bottom: Integrated density of Western blot bands was quantified. *n* = 3 independent assays. **G** Total protein abundance in DMSO-treated cells overexpressing GFP and RA-treated cells overexpressing RARγ or GFP. A significance level test was performed (relative to DMSO-treated cells overexpressing GFP). *n* = 3 independent assays. **H** RT–qPCR analysis of eukaryotic translation initiation factor-related genes in RA + GFP- and RA + RARγ-treated MuSCs. *n* = 3 independent assays. Statistical results are expressed as the mean ± SEM. n.s., not significant. *p < 0.05, **p < 0.01, ***p < 0.001.

To further investigate the mechanisms underlying RA/RAR-induced MuSC quiescence, we analyzed the expression levels of classical quiescent (Pax7) and activation (MyoD) markers upon RA treatment. To our surprise, we did not observe any difference in the mRNA expression of Pax7 and MyoD in the cells treated with RA (RA + GFP) or RA/RARs (RA + RARβ or RA + RARγ) compared to the control cells (DMSO + GFP, Figs. 3A, B). However, the mRNA expression of MyoG, the direct target of MyoD, was dramatically downregulated when cells were treated with RA (RA + GFP) and even decreased further when treated with RA/RARs (RA + RARβ or RA + RARγ) (Fig. 3C). Correspondingly, the Western blot showed that the MyoG protein was significantly decreased upon RA/RAR (RA + RARβ or RA + RARγ) treatment compared to the control (Fig. 3D). Since MyoG is known to be a canonical direct transcriptional regulatory target of MyoD [31, 32] and exemplifies MyoD activity, we examined the protein levels of MyoD and Pax7. To test whether RA affects MyoD protein expression during early activation, we incubated single fibers and the attached MuSCs with RA throughout the isolation and culture process. MyoD protein expression in the MuSCs was blocked upon RA treatment, highlighting the indispensable role of RA on the translation inhibition of MyoD during MuSCs quiescence maintaining (Fig. S3A B). Furthermore, the MyoD protein abundance of plated FISCs detected by Western blot was significantly reduced by treatment with RA or RA + RARs, while the protein level of Pax7 was not affected (Fig. 3E). However, previous study have demonstrated that MyoD intron-retained transcripts (IRTs) in quiescent MuSCs contribute to the reduced protein abundance [5]. In this study, MyoD IRTs were validated in quiescent MuSCs but hardly detectable in cells treated with DMSO + GFP, RA + GFP or RA + RARγ by semi-quantitative PCR analysis of MyoD spliced transcripts (STs) and IRTs (Fig. S3C). These results showed that MyoD mature mRNA translation, but not MyoD transcription or intron splicing, was targeted by RA signaling in MuSCs.

Next, we wanted to determine whether RA receptors are necessary for inhibiting translation. We selected RARγ and performed loss-of-function experiments since the RARγ elicted more pronounced MyoG inhibition effects than RARβ. Knocking down RARγ by RNA interference (Fig. S3D) resulted in a significant increase in MyoD protein levels compared to the control siRNA (Fig. 3F). In addition, we found that general protein synthesis in MuSCs was significantly decreased by RA treatment, and this attenuation was further strengthened by the overexpression of RARγ (Fig. 3G). Therefore, we hypothesized that RA signaling through RARγ could inhibit MyoD and protein synthesis in MuSCs and thus prevent cell activation.

Since protein synthesis is principally regulated at the initiation stage, we measured the expression levels of the translation initiation factors. Interestingly, the mRNA expression levels of the translation initiation factors in the MuSCs were not reduced in either the RARγ-overexpressing cells in vitro (Fig. 3H) or the quiescent cells in vivo (Fig. S3E). This finding suggests that the posttranscriptional regulation of translation initiation factors could be the main cause of protein synthesis inhibition in quiescent MuSCs.

### RARγ affects MyoD protein synthesis via the Akt/eIF4EBP1 signaling cascade

**Fig. 4:**
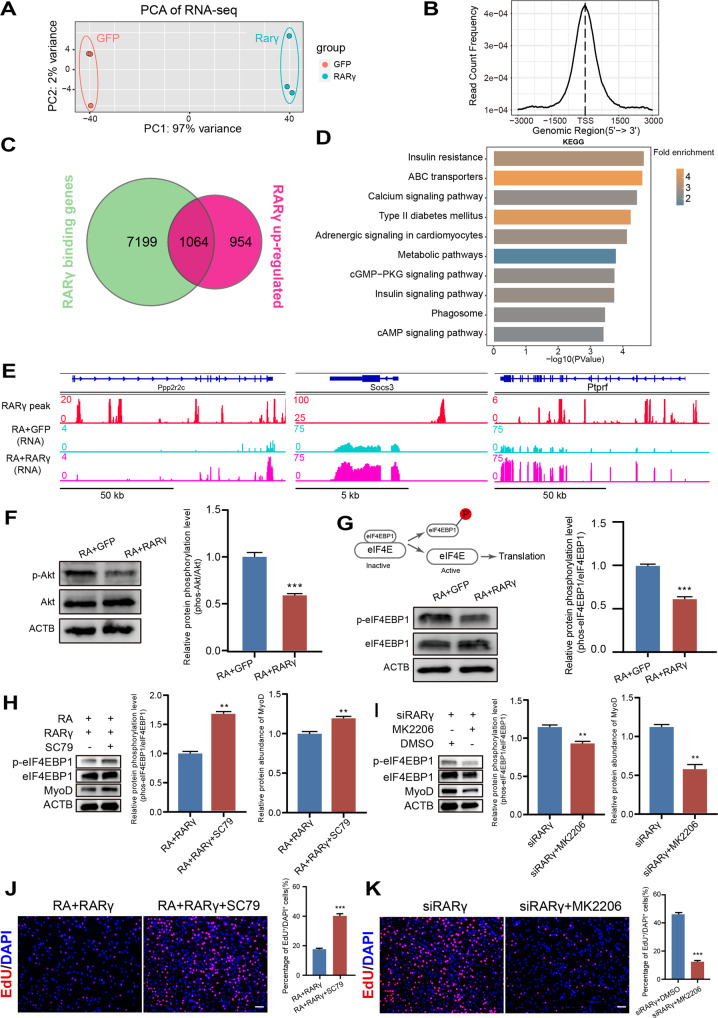
RARγ affects MyoD protein synthesis via the Akt/eIF4EBP1 signaling cascade. **A** Principal component analysis (PCA) of gene expression in MuSCs transduced with RARγ-FLAG or the GFP overexpression adenovirus. *n* = 3 biological replicate RNA-seq data/group. **B** Genomic profiles of CUT&Tag around TSSs for RARγ. **C** Venn diagram of genes whose TSSs were bound by RARγ and significantly upregulated in RARγ-overexpressing MuSCs. **D** KEGG analysis of 1064 overlapping genes in **C**. The top 10 categories are shown. **E** Integrative Genomics Viewer (IGV) track of the Ppp2r2c, Socs3 and Ptprf genes. **F** Left: western blot analysis of total Akt and phosphorylated Akt (p-Akt) in RA/RARγ- or RA/GFP-treated cells. Right: Integrated density of Western blot bands was quantified to determine the ratio of phosphorylated Akt. *n* = 3 independent assays. **G** Top left: phosphorylated eIF4EBP1 activates translation. Bottom left: western blot analysis of total eIF4EBP1 and phosphorylated eIF4EBP1 (p-eIF4EBP1) in RA/GFP- or RA/RARγ-treated cells. Right: integrated density of western blot bands was quantified to determine the ratio of phosphorylated eIF4EBP1. *n* = 3 independent assays. **H**, **I** Left: western blot of eIF4EBP1 and MyoD and phosphorylated eIF4EBP1 (p-eIF4EBP1) in cells upon treatment with an Akt agonist (SC79) or antagonist (MK2206). Right: integrated density of Western blot bands was quantified to determine the ratio of phosphorylated eIF4EBP1 and the protein expression level of MyoD. *n* = 3 independent assays. **J**, **K** EdU analysis of cells upon treatment with the Akt agonist (SC79) or antagonist (MK2206). EdU-positive MuSCs were quantified. *n* = 3 independent assays/condition, >3000 cells counted/assay. Scale bar: 50 µm in (**J**) and (**K**). Data are presented as the mean ± SEM. **p < 0.01, ***p < 0.001.

Since RA signaling mainly exerts its physiological function through the binding of the RA/RAR complex to DNA to regulate the transcription of target genes, we then tried to identify the potential downstream targets of ligand-activated RARγ that could contribute to the blockade of protein synthesis. First, the global gene expression profiles of the RA- and RA + RARγ-treated cells were obtained. PCA showed that the transcriptomes of the two types of cells (RA vs. RA + RARγ) were clearly separated, indicating that ligand-activated RARγ indeed caused widespread changes in gene expression (Fig. 4A). Next, we transduced cells with a FLAG-tagged RARγ-overexpressing adenovirus and obtained the whole genome binding profiles of activated RARγ by using CUT&Tag tools. The two biological repeats of CUT&Tag experiments showed high reproducibility and were combined for subsequent peak calling analysis (Fig. S4A). The RARγ binding sites were highly enriched around the transcription start site (TSS) regions (Fig. 4B). In addition, the motif of RARγ (RARE) was significantly enriched (−log *P*-value = 67) in CUT&Tag data (Fig. S4B). Both findings suggested that RARγ functioned as a nuclear receptor to bind and regulate gene transcription. We identified a total of 36435 conservative RARγ binding sites across the genome, and further integrated analysis revealed 1,064 genes whose TSS region was bound by RARγ and showed upregulated gene expression (Fig. 4C). Signaling pathway enrichment analysis suggested that these genes are involved in insulin resistance, calcium signaling pathways and ATP-binding cassette (ABC) transporters (Fig. 4D). Moreover, the Integrative Genomics Viewer (IGV) showed an enriched RARγ binding signal on the promoter regions of representative genes such as Socs3, Ptprf and Ppp2r2c, which are associated with the promotion of insulin resistance signaling [33–36]. Consequently, the mRNA expression levels of these genes were higher upon RARγ overexpression (Fig. 4E). For instance, protein phosphatase 2 A (PP2A) can directly dephosphorylate Akt to inhibit its activity [37, 38]. In the mRNA expression data, both Ppp2r2c and Ppp2r2b, which encode the regulatory subunit of PP2A, were significantly upregulated upon RA/RARγ treatment (Fig. S4C). In particular, the transcription start site of Ppp2r2c was also bound by RARγ (Fig. 4E), which indicates that RARγ could transcriptionally activate PP2A and, in turn, dephosphorylate and inactivate Akt.

Previous studies have shown that insulin resistance pathway genes can block Akt activity [39], stimulating translation by phosphorylating the translation repressor eIF4EBP1 [40]. In particular, only dephosphorylated eIF4EBP1 can interact with eIF4E to inhibit the assembly of the translation initiation complex, and the phosphorylation of eIF4EBP1 results in its dissociation from eIF4E and mRNA translation initiation [41]. In other words, the deprivation of insulin resistance signaling could activate Akt and in turn phosphorylate eIF4EBP1 to allow translation initiation. Therefore, we postulate that during the quiescence-to-activation transition, the cells downregulate RARγ and lose RARγ-induced insulin resistance signaling and consequently phosphorylate both Akt and eIF4EBP1 to enable translation initiation by eIF4E. Accordingly, we found that both Akt and eIF4EBP1 phosphorylation levels were significantly decreased in RA + RARγ-treated cells (Fig. 4F, G), and these decreased phosphorylation levels would block protein translation by inhibiting eIF4E.

Previous studies have demonstrated that eIF4F (the translation initiation complex composed of eIF4G and eIF4E) is not necessary for recruitment of unstructured model mRNAs to the translation machinery and eIF4G is not requiered for all translation initiation events [42, 43]. In order to confirm that the eIF4F complex was responsible for the reduction of protein synthesis of MyoD when MuSCs treated with RA/RARγ, we treated the cells with the 4EGI1 (antagonist against the interaction between eIF4E and eIF4G) and examined MyoD protein expression. As shown in Fig. S4D, cells treated with 4EGI1 showed significantly lower MyoD protein levels, indicating that MyoD translation was indeed initiated by eIF4F. However, the amount of total eIF4E and phosphorylated eIF4E (inactivated) remained constant (Fig. S4E), which suggests that the interaction between eIF4EBP1 and eIF4E could be the major regulator of eIF4F complex activity in MuSCs.

To further verify that Akt is the main effector regulated by RARγ that affects eIF4EBP1 phosphorylation and MyoD protein synthesis, we performed a series of gain- and loss-of-function experiments by manipulating Akt activity. We treated RARγ-overexpressing MuSCs with an Akt agonist (SC79) or RARγ-depleted MuSCs with an Akt antagonist (MK2206) and examined the levels of eIF4EBP1 phosphorylation and MyoD protein expression. Western blot analysis showed that in RARγ-overexpressing cells, the constitutive activation of Akt with SC79 increased both eIF4EBP1 phosphorylation and MyoD protein expression (Fig. 4H). Conversely, blocking Akt with MK2206 inhibited eIF4EBP1 phosphorylation and MyoD protein expression, even in the absence of RARγ (Fig. 4I). These results confirmed that Akt is the major effector downstream of RA signaling that regulates protein synthesis in MuSCs.

Next, we examined the cell cycle status upon Akt activation or inactivation in MuSCs. Accordingly, the Akt agonist SC79 relieved the quiescence induction caused by RA + RARγ treatment and stimulated cell proliferation (Fig. 4J). In contrast, the Akt antagonist MK2206 attenuated the cell proliferation induced by the administration of the anti-RARγ siRNA (Fig. 4K). In addition, the differentiation capacity of the MuSCs was also increased upon SC79 treatment in RA + RARγ-treated cells (Fig. S4F). Conversely, MK2206 inhibited the differentiation capacity of MuSCs (Fig. S4G).

We conclude that RARγ binds to chromatin and stimulates the transcription of target genes to inactivate Akt, leading to the dephosphorylation of eIF4EBP1 and blockage of protein synthesis. Consequently, impaired MyoD protein synthesis restrict the cell cycle re-entry of MuSCs and further myogenic differentiation, leading to a quiescent state.

### RA signaling maintains the quiescence of satellite cells both in vivo and in vitro

**Fig. 5:**
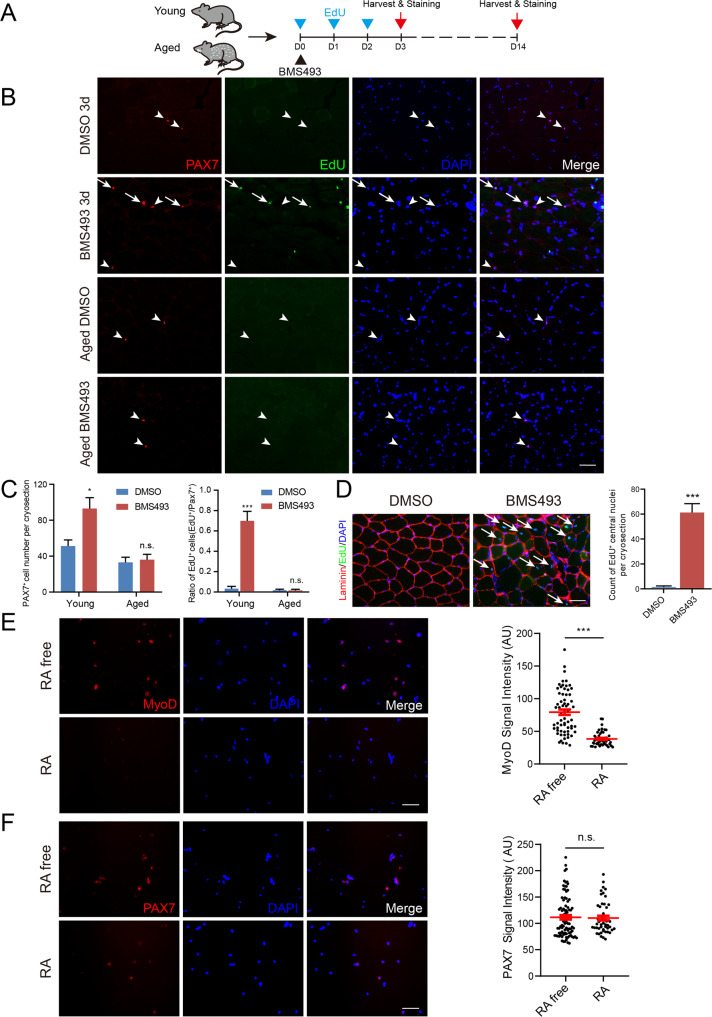
RA signaling maintains the quiescence of satellite cells both in vivo and in vitro. **A** Experimental design for the in vivo blocking of RA signaling in MuSCs. **B**, **C** EdU and BMS493 were injected as described in **A**, and cryosections of the TA muscle from young and aged mice on D3 were stained for PAX7 and EdU. Arrows represent EdU^+^/PAX7^+^ cells; arrowheads represent EdU^-^/PAX7^+^ cells. **C** Left: total number of MuSCs (PAX7^+^) per cryosection were quantified. Right: Ratio of EdU-positive MuSCs (EdU^+^/PAX7^+^) per cryosection were quantified. *n* = 3 mice/treatment, 5 cryosections/mouse, >400 PAX7^+^ cell counted/mouse. **D** Left: cryosections of the TA muscle from young mice on D14 as described in **A** were stained for Laminin and EdU. Arrows represent EdU-positive nuclei located in the center of the muscle fibers. Laminin was used to mark the interstitial space between muscle fibers. Right: EdU-positive nuclei located in the center of the muscle fiber per cryosection were quantified. *n* = 3 mice/treatment, 5 cryosections/mouse, >300 central nuclei counted/mouse. **E**, **F** Left: MyoD (left panel of E) and PAX7 (left panel of **F**) staining of cells treated with or without RA throughout the MuSC isolation process. Scatter plot representing the immunofluorescence signal intensity of MyoD (right panel of **E**) and PAX7 (right panel of **F**). *n* = 3 independent assays/condition, >100 MyoD^+^ or PAX7^+^ cells analyzed /assay. Scale bar: 50 µm in **B** and **D**, 100 µm in **E** and **F**. Data are expressed as the mean ± SEM. n.s., not significant, *p < 0.05, ***p < 0.001.

Based on the above results, we speculated that quiescent MuSCs accumulated RA substantially through the retinol metabolism pathway and constitutively activated RARγ, which in turn inhibited MyoD protein synthesis. The quiescent cells were continuously constrained in a low protein-synthesis dormant state guarded by RA signaling. Therefore, it is reasonable to hypothesize that blocking RARγ signaling in vivo can cause MuSCs to exit the quiescent state and re-enter the cell cycle, followed by fusion with neighboring myofibers. To validate this hypothesis, BMS493, an pan-antagonist against the RAR, was injected into mouse TA muscles, and EdU was administered to label MuSCs that could escape from the quiescent state (Fig. 5A). We observed EdU-positive MuSCs (EdU^+^/PAX7^+^) 3 days after BMS493 injection (Fig. 5B, C), and EdU labeling retained nuclei in the center of myofibers 14 days after injection, indicating that quiescent MuSCs proliferated and fused with neighboring myofibers (Fig. 5D). Therefore, the in-situ interruption of RA signaling without injury resulted in MuSCs escaping quiescence, highlighting the essential role of RA and RARs in maintaining MuSC quiescence. We also tested the cell cycle re-entry ability of quiescent MuSCs in aged mice to assess muscle regeneration potential. However, aged mice showed almost no EdU-positive MuSCs 3 days after BMS493 injection (Fig. 5B, C). This finding indicates that MuSCs in aged mice lose the capacity of quiescence exit upon the withdrawal of RA signaling. The diminished expression of RARs in aged MuSCs [44] could make them nonresponsive to activation signals caused by RA deprivation.

Next, we further validated the contribution of RA to the maintenance of the quiescent state of MuSCs using different applications. We forcibly activated RA signaling by adding RA to the medium throughout the normal MuSC isolation process, as this would otherwise lose RA signaling and translate MyoD abundantly. Immunofluorescence staining of the MyoD protein showed that compared with the conventional isolation process (RA free), the addition of RA during MuSC isolation effectively inhibited MyoD protein synthesis (Fig. 5E). However, PAX7 showed no significant difference with or without RA treatment during the isolation process of MuSCs (Fig. 5F), which indicated that RA, at least partially, preserved the quiescent characteristic of MuSCs. Therefore, the administration of RA could be used in various cellular applications to preserve the quiescent status of MuSCs in vitro.

Moreover, we comparatively analyzed the quiescence in MuSCs induced by a free-floating culture [45] and RA treatment. EdU analysis revealed a significant decrease in the proliferation rate of MuSCs in the free-floating culture compared to the normal adherence culture (Fig. S5A). However, the expression of classic MuSC genes, including several canonical quiescence marker genes (Pax7, Calcr), was abnormally downregulated in the free-floating MuSCs (Fig. S5B). This result indicates that free-floating cells may not necessarily enter into a genuine quiescent state in this scenario; instead, cell activity is irreversibly arrested. Accordingly, we found that the differentiation capacity was severely impaired in the free-floating treated MuSCs compared to the RA washed cells (Fig. S5C). In contrast, the RA-treated cells preserved robust capacity of proliferation and differentiation after the RA washed out (Fig. S5C, D), indicate that the RA-induced quiescent state is reversible. In conclusion, the difference in the differentiation capacity between the free-floating arrested cells and RA-treated cells suggests that RA could better preserve the native stemness of MuSCs than simply repressing cell activity.

## Discussion

In recent decades, cultured MuSCs have been widely used to study muscle cell proliferation and differentiation to uncover the molecular mechanisms that could direct muscle development and regeneration events in vivo. However, recent studies have shown that epigenetic modifications, such as the posttranscriptional regulation and protein modification of MuSCs are dramatically altered during their isolation from the native environment inside the body [5, 9], indicating that MuSCs may behave differently in vitro and in vivo. Therefore, cultured MuSCs may not accurately replicate natural muscle development and regeneration events. To preserve the fidelity of quiescent and activated MuSCs in vivo, fixation by PFA perfusion was utilized to preserve the genuine state of MuSCs before isolation, and high-quality RNA was obtained without overfixation for library construction and gene expression analysis [46]. Subsequent transcriptome analysis showed that quiescent MuSCs highly expressed the quiescent-specific marker genes Pax7 and CD34 [47, 48] and Notch pathway genes such as Rbpjl and Heyl. These genes are involved in suppressing MyoD expression to maintain MuSC quiescence [49, 50]. In addition, the stress index genes induced in the conventional isolation process without fixation [8] were minimally expressed in our quiescent and activated cells in vivo. These data demonstrated that the current transcriptome adequately preserved the native signatures of MuSCs in situ and thus enabled us to uncover the molecular mechanisms that control the bona fide quiescent status.

MuSCs can be activated by various extrinsic and intrinsic stimuli, but whether conserved signaling or multiple complementary mechanisms can control quiescence-to-activation transitions remains unclear. We used different means to activate MuSCs in vivo to explore their true quiescence identity. The commonly used myotoxic agents (e.g., cardiotoxin) mainly damage myofibers, and the affiliated MuSCs are activated to repair damaged fibers [51–53]. Freeze injury destroys all cells, including MuSCs, in the injury zone, and muscle regeneration is mainly accomplished by the inward migration of MuSCs from the outside area of injury [54]. Muscle hypertrophy-induced MuSC activation is independent of muscle injury and regeneration, which results in a functional overload of synergist muscle and produces minimal damage to cellular structures [55]. The live MuSCs freshly isolated with the traditional method also showed a high degree of activation [9]. To prevent interference from each of the specific injury models or isolation processes, we combined multiple transcriptomes from MuSCs activated by distinct methods and found that retinoic acid signaling was conserved in quiescent cells but not in any of the enriched activated cells.

The metabolism of retinol or RA has been implicated in maintaining quiescence in several cellular systems. For example, dormant hematopoietic stem cells highly express RA-associated genes, and RA treatment enables the cells to antagonize induced activation by limiting protein translation and reactive oxygen species levels [13, 56]. Retinoic acid signaling also confers quiescence and stemness properties to various cancer stem cells, such as colon carcinoma cells and leukemic stem cells [57, 58]. Conversely, embryonic stem cells, cancer stem cells and MuSCs all lose Adh1 expression during cellular activation [59, 60] and thus cannot metabolize retinol to RA to maintain quiescence. Consistent with this model, RA treatment inhibited the proliferation and differentiation of both hematopoietic stem cells and MuSCs [13, 28, 61]. On the other hand, RA is also well known as an inductor of differentiation in several cell systems, such as blood cells and specific leukocytes [62, 63]. The Janus face of RA in promoting either stemness or differentiation may be explained by different cell types, cellular states, and the expression of specific RARs [64]. For example, RARγ functions as a tumor suppressor in colorectal cancer. Silencing RARγ promotes colorectal cancer cell growth, migration, invasion, and metastasis [65]. RARβ possesses many functional characteristics of a tumor suppressor in breast cancer cells; however, it promotes the growth and progression of mammary epithelial tumors [66]. A better understanding of the inconsistent regulatory mechanisms of cell stemness and differentiation by RA may improve the therapeutic applications of this factor in regenerative medicine and as a tumor treatment.

Quiescent stem cells usually show low-level energy metabolism features, such as reduced mRNA transcription and protein synthesis [67–69]. Accordingly, we observed much lower protein synthesis in RA/RARγ-treated MuSCs. However, we were intrigued by the stable mRNA transcription of MyoD in both injury-activated MuSCs in vivo and RA/RARγ-treated MuSCs in vitro. This result suggests that the transition of MuSCs from a quiescent to an activated state in vivo does not require the transcriptional upregulation of MyoD [5]. However, artificially enhancing MyoD expression can also promote the activation of MuSCs [70]. Recent studies have reported that the transcription-independent inhibition of MyoD protein synthesis can maintain MuSC quiescence, which could be achieved by attenuating the Dek excision of MyoD mRNA introns [5] or enhancing the binding of Staufen1 to the 3'UTR of MyoD mRNA [71]. In parallel, we found that RA-activated RARγ attenuated protein synthesis by inhibiting eIF4EBP1 phosphorylation via the regulation of Akt activity and contributing to the blockage of MyoD protein synthesis. Theoretically, the MyoD protein synthesis suppressed by the RA signaling is independent of intron splicing and this translation inhibition would prevent any residue MyoD protein production from the available mature MyoD mRNAs that were properly spliced. Therefore, the abrogation of MyoD protein in quiescent MuSCs could be achieved by multiple layers of regulations, but the detailed complementary mechanisms require further study.

As described above, quiescent MuSCs are a highly regulated homeostatic process of low protein synthesis. Protein synthesis is tightly controlled by the translation initiation complex, in which eIF2α is an essential component, and its phosphorylation is required for MuSC quiescence and self-renewal [72]. In addition, the phosphorylation of eIF4EBP1 has been reported to contribute to translation inhibition and the maintenance of stemness in adult stem cells such as hematopoietic stem cells and neural stem cells [73, 74], and the function of eIF4EBP1 in MuSCs was first discovered in this study. The integrated analysis of the RARγ chromatin binding profile and the quiescence transcriptome revealed RARγ-targeted genes that contribute to Akt and eIF4EBP1 dephosphorylation to inhibit translation initiation. This RARγ-directed translation repression mechanism is rescued by the Akt agonist SC79, providing additional supporting evidence that the RARγ/Akt/eIF4EBP1 signaling cascade maintains MuSCs in the quiescent state.

In summary, we found that RA and RARγ maintained MuSC quiescence by attenuating Akt and eIF4EBP1 phosphorylation to hinder protein synthesis. Typically, RA/RARγ decreases MyoD protein synthesis independent of transcriptional alterations, resulting in MuSC proliferation and differentiation being blocked and keeping them in a quiescent state. This finding provides additional insight into the regulatory mechanisms of muscle stem cell quiescence maintenance and stemness.

## Experimental procedures

### Animals

Rosa26^tdTomato/tdTomato^ mice were crossed with Pax7^creER/creER^ mice to obtain the Pax7^creER/+^; Rosa26 ^tdTomato /+^ mice used for inducing injury and isolating MuSCs. Pax7^creER^ mice (Stock No.#017763) and Rosa26 ^tdTomato^ mice (Stock No. #007914) were obtained from The Jaxson Laboratory (Farmington, CT).

All 8–12-week-old C57BL/6 wild-type and Pax7^CreER/+^;Rosa26^Tdtomato/+^ young mice and 24-month-old aged mice were housed in a pathogen-free environment at 24–26°C and on a 12 h:12 h light/dark cycle under Huazhong agricultural university veterinary staff supervision.

All experimental protocols for mice used in this study were performed in accordance with the guidelines of the Animal Care and Ethics Committee of Huazhong Agricultural University. C57BL/6 mice were obtained from Laboratory Animal Center of Huazhong Agricultural University. Males and females were randomly allocated to experimental groups and no blinding method was used. There was no animal exclusion criteria.

### Animal models

Tamoxifen (SIGMA, T5648) dissolved in corn oil (20 mg/ml, 5 μl/g body weight) was injected intraperitoneally once every 24 h for a total of five consecutive days. There is a 5-day waiting period between the final injection and the following analysis.

Different means (cardiotoxin injury, freeze injury, synergist ablation) to activate the MuSCs were performed, and the time points of tissue havest and cell isolation were determined according to previous reports [55, 75] to achieve the maximum cell activation. To induce muscle cardiotoxin injury, 50 μl of 10 μM cardiotoxin (MCE, HY-P1902A) was injected intramuscularly into the tibialis anterior (TA) muscle in the both legs of respiratory anesthetized (3% isoflurane and 1.5 L of O_2_ per minute) mice using PV830 Pneumatic PicoPump (World Precision Instruments, Sarasota, FL, USA). TA muscle and proliferating MuSCs were isolated 3 days after cardiotoxin injection.

Skeletal muscle freeze injury was induced by exposing skeletal muscle to an extremely cold probe, and results in a robust degenerative response [54]. Briefly, A 6-mm-diameter steel rod probe was precooled in liquid nitrogen. Mice were respiratory anesthetized and TA muscles were exposed. Then, place the probe tip on the mid-belly of the TA and press firmly for 10 s. After the frozen-injured area change from white to pink color, repeat the above rapid freeze–thaw three times to kills all cells in the zone of injury. TA muscle and proliferating MuSCs were harvested 4 days post injury.

Skeletal muscle hypertrophy was induced by mechanical overload of the plantaris muscle by synergist ablation (SA) as described previously [55]. Briefly, for SA model, approximately half of the proximal portion of the gastrocnemius and the soleus of mice were excised. Following incision closed with surgical sutures, the mice were returned to a clean cage and monitored for recovery from anesthesia. At 7 days after surgery, the mice were euthanized and the plantaris muscles were harvested.

For BMS493 treatment mice, 50 μl of 5 mM BMS493 (MCE, HY-108529) in 10% DMSO (diluted in corn oil) was injected to TA muscle of adult and aged wild type C57BL/6 mice along with intraperitoneal injection of EdU for three consecutive days. 10% DMSO in corn oil were injected to the TA muscle of contralateral legs as control. TA muscle were harvested and stained 3 days or 14 days after BSM493 injection. Cells in whole area of TA muscle section were counted after staining.

### Satellite cell isolation via fluorescence activated cell sorting (FACS)

MuSCs in vivo (quiescent, CTX injury, freeze injury and mechanical overload) were isolated according to the protocol described previously with slightly modification [5]. A total of 100k quiescnet cells can be obtained per adult mouse limb muscles. Approximately 30–40k MuSCs can be obtained in CTX injury, freeze injury and mechanical overload mice.To maintain a consistent number of cells, we used a uniform 30k cells to construct the transcriptome library.

In brief, mice were first perfused with 50 mL pre-chilled PBS using injection pump at speed of 10 mL/min from the left cut ventricle to the right cut atrium, and then fixed with 50 mL pre-chilled 0.5% PFA (diluted in PBS). Afterward, 50 mL pre-chilled 2 M glycine (diluted in PBS) was perfused to quench fixation. Hindlimb muscles from the mouse were dissected and minced. Minced muscles were digested with collagenase II (Worthington, LS004177, 2000 U/mL) and dispase II (ROCHE, 4942078001 1 U/mL) in DMEM for 120 min. For freshly isolated MuSCs, minced muscles were digested with collagenase II (500 U/mL) in DMEM for 90 min. Digest suspension was diluted with DMEM to 100 mL per mouse. After filtered with 40 μm cell strainer, the digest suspension was centrifuged at 50 g for 10 min at 4 °C. Discarded the pellet and centrifuged the supernatant at 600 g for 10 min at 4 °C for three times. Cell pellet was suspended with DMEM to 1–2 mL and sorted using a fluorescence activated cell sorter (BD, BD FACS Aria II) equipped with 355 nm, 445 nm, 488 nm, 561 nm, 640 nm lasers. The machine was carefully optimized for purity and viability.

### Single fiber explants isolation

Extensor digitorum longus (EDL) muscles were carefully dissected and digested with collagenase II (Worthington, LS004177, 1000 U/mL) in DMEM in a 37 °C water bath for 75 min. Single fibers were obtained by flushing the EDL muscle with wash medium (10% FBS in DMEM) using Glass Pasteur Pipette in a horse serum coated 6-cm cell culture dish (NEST Biotechnology). Single fibers were placed in single fiber culture medium (20% fetal bovine serum, 1% chicken embryo extract, 1% Penicillin–Streptomycin). Culture single fibers in a CO_2_ incubator at 37 °C [5, 76]. DMSO or RA was constantly supplied throughout the isolation and culture process. Fibers were fixed and immunostained 6 h after EDL isolation for PAX7/MyoD staining, and 24 h after EDL isolation for PAX7/Mcm2 staining.

### Cell culture and transfection

MuSCs were cultured on Matrigel-coated (BD, 356234) plates with growth medium containing 20% FBS, 0.5% CEE (Genimi, 100-163 P), 1% GlutaMax (Gibco, 35050061), 1% NEAA (Gibco, 11140-050), 1% AA (Gibco, 15140-122), RPMI 1640 (Gibco, C11875500BT), 2.5 µg/µl bFGF (Gibco, 13256-029) at 37 °C in 5% CO_2_. The differentiated cells were obtained through culturing in differentiation medium (5% Horse Serum, 1% AA, DMEM) at 37 °C in 5% CO_2_ when cell confluence reaches 80%-90%. Cells were transfected with 100 nM (final concentration) siRNA anti-RARγ/NC (Guangzhou Ribobio) or 1 μg plasmid per well (12 well culture plate) using jetPRIME (Polyplus, 101000046) according to the user manual. After incubating for 4–6 h, cells were washed twice with pre-warmed PBS and cultured in growth medium. Transfection efficiency was measured using a vector expressing GFP or negative control oligo conjugated with FAM after 24 h transfection.

MuSCs induced quiescence in vitro through free-floating myospheres was described previously [45]. In brief, MuSCs were seeded on 90 mm bacterial dishes in the MuSCs growth culture medium with the addition of 0.3% methylcellulose (Sigma, 09967). Then, MuSCs do not adhere to dishes and join together in aggregates. After 7 days culturing, MuSCs aggregates were trypsinized and pulse-labeled with EdU for 2 h. MuSCs were then centrifuged with 13,000 rpm for 5 min and the cell pellets were embedded in optimal cutting temperature compound (OCT, cell path, KMA-0100-00A). 20 μm frozen sections were prepared and stained with EdU. In parallel, MuSCs from trypsinized aggregates were re-adhered on pre-coated dishes and induced differentiation for 48 h.

### Immunofluorescence staining

TA and plantaris muscles cryosections were fixed with 4% PFA for 10 min. After antigen retrieval with citrate buffer (PH 6.0) using a pressure cooker in high pressure mode for 10 min, muscles cryosections were blocked with blocking solution consisting of 1% Triton X-100 and 5% BSA (beyotime, ST023) in PBS for 2 h, followed by incubation with primary antibodies diluted in blocking solution at 4 °C overnight. After washed with PBST (0.1% Triton X-100 in PBS) for three times, the cryosections were incubated with secondary antibodies diluted in blocking solution at room temperature for 2 h. Stained muscles were counterstained with DAPI and mounted with mounting medium.

For cultured cells and single fibers, cells or single fibers were fixed with 4% PFA for 10 min, and then permeated with 0.5% Triton X-100 for 15 min. After blocking cells or single fibers with blocking solution for 1 h at room temperature, incubated cells or single fibers with primary antibodies at 4 °C overnight. Cells or single fibers were washed with PBS for three times, and incubated with fluorophore-labeled secondary antibodies for 1 h, washed and counterstained with DAPI.

### Antibodies

Antibodies used in this study are: anti-PAX7 (DSHB, deposited by Kawakami, A.), anti-MF20 (DSHB, deposited by Fischman, D.A.), anti-Akt (Proteintech, 10176-2-AP), anti-phosphorylated-Akt (CST, 4060), anti-eIF4E (ZENBIO, 384193), anti-phosphorylated-eIF4E (CST, 9741), anti-eIF4EBP1 (ZENBIO, R24197), anti-phosphorylated-eIF4EBP1 (CST, 2855), anti-MyoD (Santa cruz, sc-32758X), anti-MyoD(Active motif, 39991), anti-Mcm2(Abcam, ab4461), anti-Myog (Santa cruz, sc-12732), anti-ACTB (Abclonal, AC026), anti-FLAG (Abclonal, AE005), anti-mouse secondary antibody conjugated with Alexa Fluor 488 (Invitrogen, A-21202), anti-mouse secondary antibody conjugated with Alexa Fluor 555 (Invitrogen, A-31570), anti-rabbit secondary antibody conjugated with Alexa Fluor 488 (Invitrogen, A-21206), anti-rabbit secondary antibody conjugated with Alexa Fluor 555 (Invitrogen, A-31572), HRP anti-mouse secondary antibody (beyotime, A0216), HRP anti-rabbit secondary antibody (beyotime, A0208).

### EdU incorporation analysis

5-ethynyl-2'-deoxyuridine (EdU, Invitrogen, E10187) was dissolved in sterilized PBS at 2.5 mg/mL and stored at −20 °C. The stock EdU solution was diluted with PBS (0.5 mg/mL) and injected intraperitoneally into mice at 5 mg/kg body weight 12–24 h before analysis. For cultured cells, cells incubated with 50 μM EdU dissolved in culture medium 2 h before analysis.

Muscles cryosections and cultured cells were fixed with 4% PFA for 10 min, and then permeated with 0.5% Triton X-100 for 15 min. After washed with PBS, cells were incubated with EdU staining buffer(100 mM Tris, 1 mM CuSO4, 10 mM fluorescent azide, and 100 mM ascorbic acid) for 30 min at room temperature. For EdU incorporation analysis on FISCs, EdU (50 μM) was incubated throughout the isolation process. Stained muscles cryosections and cultured cells were counterstained with DAPI.

### Cell cycle analysis

Cell cycle analysis were performed using DNA content quantitation assay kit (Solarbio, CA1510) according to the manufacturer’s instructions. In brief, cells derived from trypsin dissociation of the aggregates were fixed in cold 70% ethanol overnight, then washed, and incubated with 100 μl RNase A at 37 °C for 30 min. Incubate with 400 μl PI staining buffer at 4 °C for 30 min. Cells without PI treatment were collected as negative control. Flow cytometric analysis was performed on a fluorescence activated cell sorter (BD, BD FACS Aria II) using 488 nm lasers and PE-Texas red-A channel. In-built function of FlowJo (version 10.8.1) software was used to analyze the flow cytometric data. Doublets were discriminated by means of gating.

### RNA isolation and smart-seq2 library construction

For cultured MuSCs, RNA was extracted using TRIzol reagent (Simgen, 5301100). The RNA quality and concentration were determined using NanoDrop 2000 (Thermo, USA). For PFA fixed cells in vivo, RNA from 30k cells was extracted using a QIAGEN RNeasy FFPE Kit (Qiagen, 73504) according to the manufacturer’s instructions. RNA-seq library for fixed cells were constructed according to the protocol published previously [77]. In brief, 2.3 μL RNA was mixed with 1 μL 10 μM oligodT and 1 μL 10 mM dNTP, and then incubated at 72 °C for 3 min. RNA solution was mixed with RT reaction mixture (SuperScript II reverse transcriptase 100 U, RNAse inhibitor 10 U, Superscript II first-strand buffer 2 μL. DTT 5 mM, Betaine 1 M, MgCl_2_ 6 mM, TSO 1 μM, Nuclease-free water). The library PCR product was purified with KAPA Ampure beads (KAPA, KK8001). A total of 50 ng of PCR product was tagmented and enriched using TruPre DNA Library Prep Kit (Vazyme, TD501-01). First-strand and tagmented DNA library size distribution were checked by Agilent 2100 bioanalyzer. For all samples, cDNA library with peak size of 1.5-2.0 kb and tagmented DNA with a broad peak size of 300-800 bp were accepted.

### Western blot

Cells were rinsed twice with pre-chilled PBS. To prepare cell lysates, cells were scraped and lysed using RIPA lysis buffer (beyotime, P0013B) with PMSF (beyotime, ST506) and Sodium Orthovanadate (beyotime, ST1650) on ice for 30 min. The lysate was centrifuged at 13,000 rpm at 4 °C for 15 min and then 5X protein loading buffer (epizyme, LT101S) was added to the lysates prior to their full denaturation in 100 °C heating blocks for 10 min. Protein concentration was measured by BCA Protein Assay Kit (beyotime, P0012S) according to the user manual. A total of 30 μg of protein was electrophoresed on SDS/PAGE gel and transferred to a PVDF membrane (Millipore, ISEQ00010). The membrane was blocked in 5 % bovine serum albumin for 2 h and then incubated with primary antibodies at 4 °C overnight. The membranes were washed with 0.5% TBS-Tween20 and then incubated with goat anti-mouse or anti-rabbit secondary antibodies conjugated with western chemiluminescent HRP substrate for 2 h. The membranes were washed and exposed using a GE LAS 4000 imaging system (GE, USA). The captured images were analyzed using ImageJ software (version 1.8.0). The protein level of whole cell lysates normalized against the expression of β-actin.

### Real-Time PCR

500-1000 ng of the total RNA was reverse transcribed into single-stranded cDNA using an RT kit (Novoprotein, E047-01B), involving two steps as follows: 42 °C for 5 min, followed by 37 °C for 15 min. qPCR was performed using a Hieff qPCR SYBR Green MIX (Yeasen, 11201ES08), in triplicate, on a Bio-Rad Touch CFX384 (Bio-Rad, USA). The qPCR program was as follows: 95 °C for 30 s, followed by 40 cycles of 5 s at 95 °C and 40 s at 60 °C. The relative mRNA expression level was normalized to that of Gapdh. The 2^-ΔΔCT^ algorithm was employed to estimate the relative expression level of each gene.

### Expression vector construction, adenovirus package and CUT&Tag

To create RARβ/RARγ/Adh1 expression vector, RARβ/RARγ/Adh1 coding sequences was amplified and cloned into pEGFP-C1 (Youbio, VT1118) using restriction enzyme of NheI (Thermo Scientific, ER0971) and BamHI (Thermo Scientific, ER0051) to replace EGFP sequence. Homologous recombination of RARβ/RARγ/Adh1 PCR fragments and vector plasmid were performed using homologous recombination mix (ABM, E086) according to the user manual. Recombinated expression vectors were transformed into Fast T1 competent cells (Vazyme, C505-03) and plated on agarose LB medium overnight. Bacteria clones were cultured in liquid LB medium for 4–6 h and genotyped with PCR. Sequences of expression vectors were validated by sanger sequencing. Plasmids were extracted using E.Z.N.A.® Endo-Free Plasmid Mini Kit II (OMEGA, D6950-01) according to the user manual.

To package RARγ adenovirus, RARγ coding sequence and flag-tag sequence were amplified by PrimeSTAR GXL DNA Polymerase (Takara, R050Q) and cloned into pADV-CAS9-T2A-GFP vector (HedgehogBio, HH-CAS-056) using restriction enzyme of EcoRI (Thermo Scientific, ER0071) and BshtI (Thermo Scientific, ER1461). A total of 8 μg adenovirus vector whose CAS9 sequence was replaced by coding sequence of RARγ (Padv-RARγ-FLAG) transfected together with pBHGlox(delta)E1-3Cre vector (LMAI Bio, LM2214) into 293 A cells in a 2:1 ratio using Jetprime for 4–5 h. 293 A cells were cultured with 5% FBS of DMEM medium for about 7 days and ~50% cells dead at that time. Centrifuge 293 A cells with the medium at 1000 rpm for 5 min, then freeze and thaw the cell pellet with 100–200 μL DMEM five times in liquid nitrogen and a 37 °C water bath. Centrifuge at 4000 rpm for 5 min and then aliquot the supernatant containing adenovirus at −80 °C.

MuSCs were pre-treated with 1 μM RA or 0.1% DMSO for 24 h before adenovirus infection. The adenovirus supernatant was mixed with 8 μg/mL polybrene (solarbio, H8761). After 8–12 h incubation, replace the virus containing medium with fresh proliferation or differentiation medium with 1 μM RA or 0.1% DMSO. 24 h after virus infection, cells were counted and 100,000 cells were collected for CUT&Tag library construction using Hyperactive In-Situ ChIP Library Prep Kit for Illumina (Vazyme, TD901-01) according to the manufacturer’s instructions.

### Cell treatment and drugs

For MuSCs solely treated with drugs (Figs. 2A, S2D, E, D), 1 μM RA, 0.1% DMSO or 5 μg/mL 4EGI1 (Selleck, S7369) were added to the growth medium when sorted cells plated down. EdU incorporation analysis and Western blot were performed 24 h after treatment. After a further 24 h of culture in differentiation medium, the differentiation capacity was evaluated.

For MuSCs administrated with drugs and expression plasmids (Figs. 2B–F, 3A–E, G, H, 4F, G, E, S2J, S3C), sorted MuSCs were pre-cultured with growth medium in the presence of 1 μM RA (sigma, R2625), 10 μM Retinol (sigma, R7632) or 0.1% DMSO for 24 h before transfection. EdU incorporation analysis and Western blot were performed 24 h after transfection. After a further 24 h of culture in differentiation medium, the differentiation capacity was evaluated.

For MuSCs solely transfected with expressioin plasmid or anti-RARγ/NC siRNA (Figs. 3F, S2G–I and S3D), cells were pre-cultured in growth medium after FACS. RT-qPCR and Western blot were performed 24 h after transfection.

For RARγ rescue experiments (Figs. 4H–K, S4F, G), sorted MuSCs were pre-cultured for 24 h before transfection with growth medium in the presence of 1 μM RA (sigma, R2625) or 0.1% DMSO. Then 10 μM SC79 (MCE, HY-18749) or 10 μM MK2206 (MCE, HY-10358) were added to the medium 4 h after the cells were transfected with RARγ overexpression plasmid or anti-RARγ siRNA, respectively. 24 h after transfection, cells were subjected to EdU incorporation analysis or induced differentiation with medium containing 5% horse serum in DMEM for 24 h.

### Bioinformatics analysis

#### RNA sequencing analysis

HISAT2 [78] was used to align sequencing reads from each sample to the reference genome of mm10 (https://hgdownload.soe.ucsc.edu/goldenPath/mm10/bigZips/). The reference genome annotation file was downloaded from GENCODE (https://www.gencodegenes.org/mouse/release_M1.html). Reads mapping efficiency were supplied in supplementary table s1 for quiescent vs. activated and RARγ overexpressed MuSCs. FeatureCounts [79] was used to count the reads in protein-coding genes. DEseq2 [80] was used to identify differential expression genes. Annotated protein-coding genes showing |log2FoldChange | (|log2FC | ) ≥ 1 and *p*-value < 0.05 were considered to be differentially expressed genes.

#### Kyoto Encyclopedia of Genes and Genomes (KEGG) analysis

KEGG pathway analysis was implemented in the DAVID Bioinformatics Resources 6.8 version [81] (https://david.ncifcrf.gov/). The lists of gene symbols were submitted to the website. The pathways that involved the genes lists were retrieved from the KEGG pathway data base. Cut-off criteria was *p*-value < 0.05. Finally, the visualization analysis of the pathway was performed using R.

#### CUT&Tag data analysis

CUT&Tag data analyses were performed according to the ENCODE transcription factor processing chip-seq pipeline2 (https://github.com/ENCODE-DCC/chip-seq-pipeline2) with parameter of pipeline_type: “ tf”. Genome sequence file and genomic annotation file are similar to RNA-seq data analysis. The R package ChIPseeker [82] was used to identify the nearest genes around the peak and annotate genomic regions of peaks. DeepTools was used for PCA analysis, correlation analysis and assessing the enrichment profiles of RARγ CUT&Tag data. All alignment results were then converted to coverage bigwig files and normalized to the corresponding input using deepTools. The bigwig formats visualized using the Integrative Genomics Viewer (IGV) software. Reads mapping and peak calling results were supplied in supplementary table s2. Motif analysis was performed using Hommer software (v4.11) [83].

#### Quantification and statistical analysis

qPCR results are presented as mean ± SEM. Statistical comparison between two groups was performed by two-tailed unpaired Student’s *t*-test. Multi-group comparisons were performed with one-way or two-way ANOVA test followed by Bonferroni post hoc test using GraphPad Prism version 8.0 (GraphPad Software, Inc.). Immunoblot analysis is shown as mean ± SEM after densitometric image analysis with ImageJ software (National Institute of Mental Health, Bethesda, MD, USA). Comparison of RNA expression levels of eukaryotic translation initiation factors in quiescent and different injury models were performed with paired Wilcoxon test using R. In figures, asterisks denote statistical significance *P < 0.05, **P < 0.01, ***P < 0.001. No statistical methods were used to predetermine the sample size. The variance was similar between the groups that were being statistically compared.

## Supplementary information

supplementary figures and figure legends

Reproducibility checklist

Original western blots

Supplementary table1 Summary of RNA-seq data

Supplementary table2 Summary of CUT and TAG data

Change of authorship request form

## Data Availability

Data generated have been deposited in NCBI data base: PRJNA822511.
