# Supplementary material for: Retinoic acid and RARγ maintain satellite cell quiescence through regulation of translation initiation
Source: Cell Death Dis. 2022 Sep 29;13(9):838. doi: 10.1038/s41419-022-05284-9 (PMC9522790; doi:10.1038/s41419-022-05284-9)
Supplement: Supplementary file 1 — supplementary figures and figure legends [file 41419_2022_5284_MOESM1_ESM.docx]

### Supplementary figures and figure legends


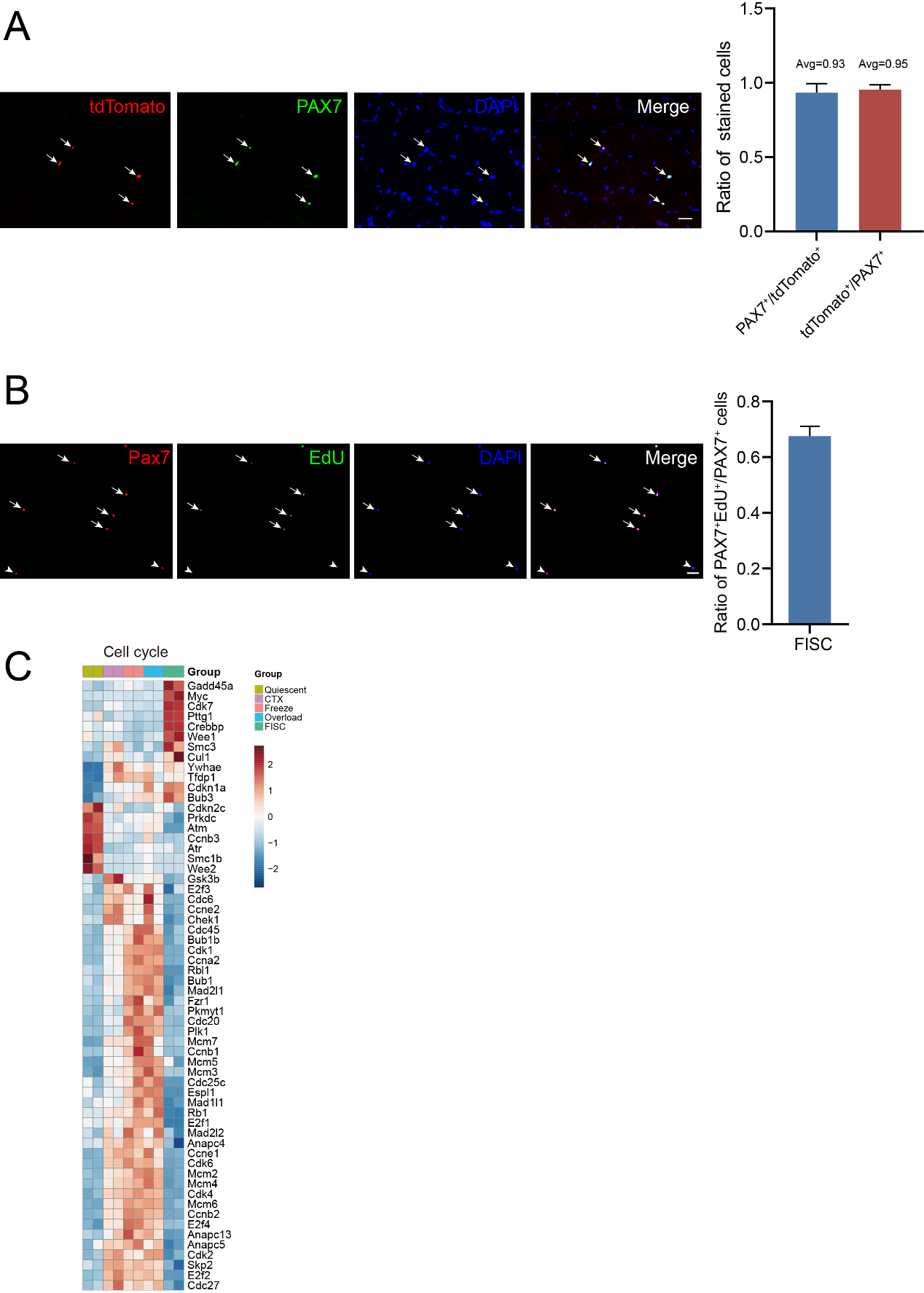


##### Fig. S1: Retinol metabolism is enriched in quiescent satellite cells, related to Fig. 1.

(A) Left: Representative pictures showing tdTomato and PAX7 staining in Pax7^creER/+^; Rosa26^tdTomato/+^ mice after tamoxifen administration. Arrows represent tdTomato^+^/PAX7^+^ cells. Right: Quantification the ratio of PAX7^+^/tdTomato^+^ or tdTomato^+^/PAX7^+^ MuSCs. n=3 mice, >200 cells counted/mouse.

(B) Representative pictures showing PAX7 and EdU staining of freshly isolated MuSCs (FISCs). Arrows represent EdU^+^/PAX7^+^ cells, arrowheads represent EdU^-^/PAX7^+^ cells. Right: Quantification the ratio of EdU^+^/PAX7^+^ MuSCs. n=3 mice, >200 cells counted/mouse.

(C) Expression heatmap of cell cycle genes.

Scale bar: 50 µm in (A) and (B). Statistical results are expressed as the mean ± SEM.


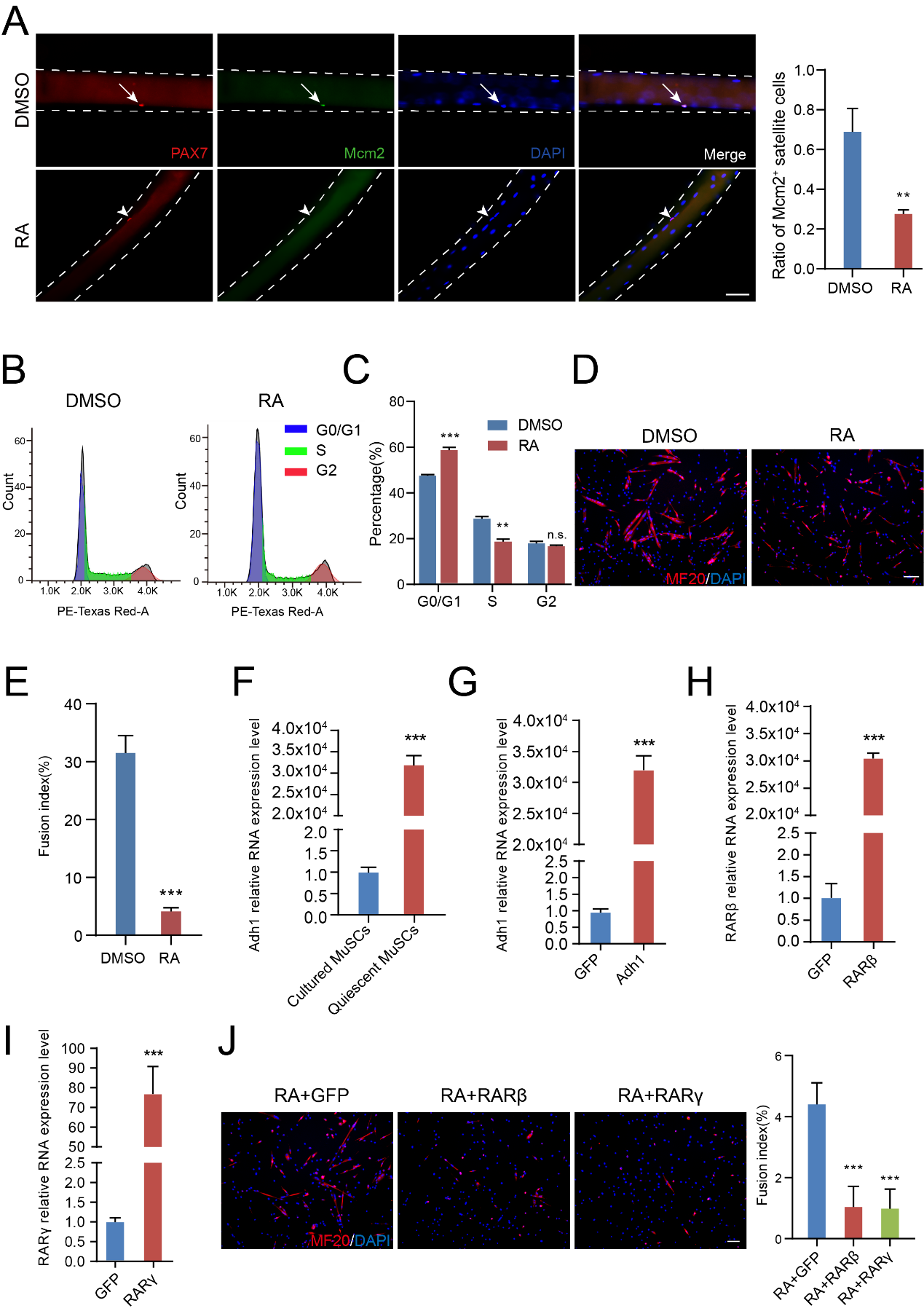


##### Fig. S2: Retinoic acid and RARγ inhibit satellite cell proliferation and differentiation, related to Fig. 2.

(A) Fiber-associated MuSCs were treated with RA or DMSO (control) to inhibit cells proliferation. Single fibers from extensor digitorum longus (EDL) muscle were incubated with DMSO/RA throughout the isolation and culture process. Left: Fiber-associated MuSCs were stained with PAX7 and Mcm2 24 h after EDL isolation. Arrow represents Mcm2^+^/PAX7^+^ cell, arrowhead represents Mcm2^-^/PAX7^+^ cell. Right: The proportion of Mcm2-positive fiber-associated MuSCs (Mcm2^+^/PAX7^+^) were quantified. n = 89 fiber-associated MuSCs.

(B) Representative images of cell cycle analysis of freshly isolated MuSCs treated with DMSO or RA for 24 h. The propidium Iodide (PI) stained (PE-Texas Red-A) nuclei of the MuSCs were analyzed for cell cycle proportions. The G0/G1 proportion is identified by the peak with single copies of DNA (n) and shows lower PI staining (around 2.0k). The S phase ranges from 2.0 to 4.0k, as cells will contain a range of DNA from n to 2n. Cells in G2 will have a peak double to that of G1 as they have diploid nuclei (2n).

(C) Bar plot quantified the percentage of cells in different cell cycle stages. n = 3 independent assays/condition, 20,000-30,000 cells/assay.

(D) DMSO or RA treated cells were induced differentiation and stained with MF20.

(E) Fusion index was calculated by quantifying the proportion of nuclei inside myotubes (3 or more nuclei) with the respect to the number of total nuclei. n=3 independent assays/condition, >1,500 cell nuclei counted/assay.

(F) RT-qPCR analysis of *Adh1* gene in the in vivo quiescent and in vitro cultured MuSCs in normal growth medium. Expression levels were normalized with Gapdh. n=3 independent assays.

(G-I) RT-qPCR analysis of overexpression efficiency for *Adh1* (G)*, RARβ* (H) and *RARγ* (I) gene. Expression levels were normalized with Gapdh. n=3 independent assays.

(J) Left: RA treated cells overexpressing GFP, RARβ or RARγ were induced differentiation and stained with MF20. Right: Fusion index was calculated by quantifying the proportion of nuclei inside myotubes (3 or more nuclei) with the respect to the number of total nuclei. n=3 independent assays/condition, >1,500 cell nuclei counted/assay.

Scale bar: 50 µm in (A), 100 µm in (D) and (J). Statistical results are expressed as the mean ± SEM. n.s., not significant. **p<0.01, ***p<0.001.


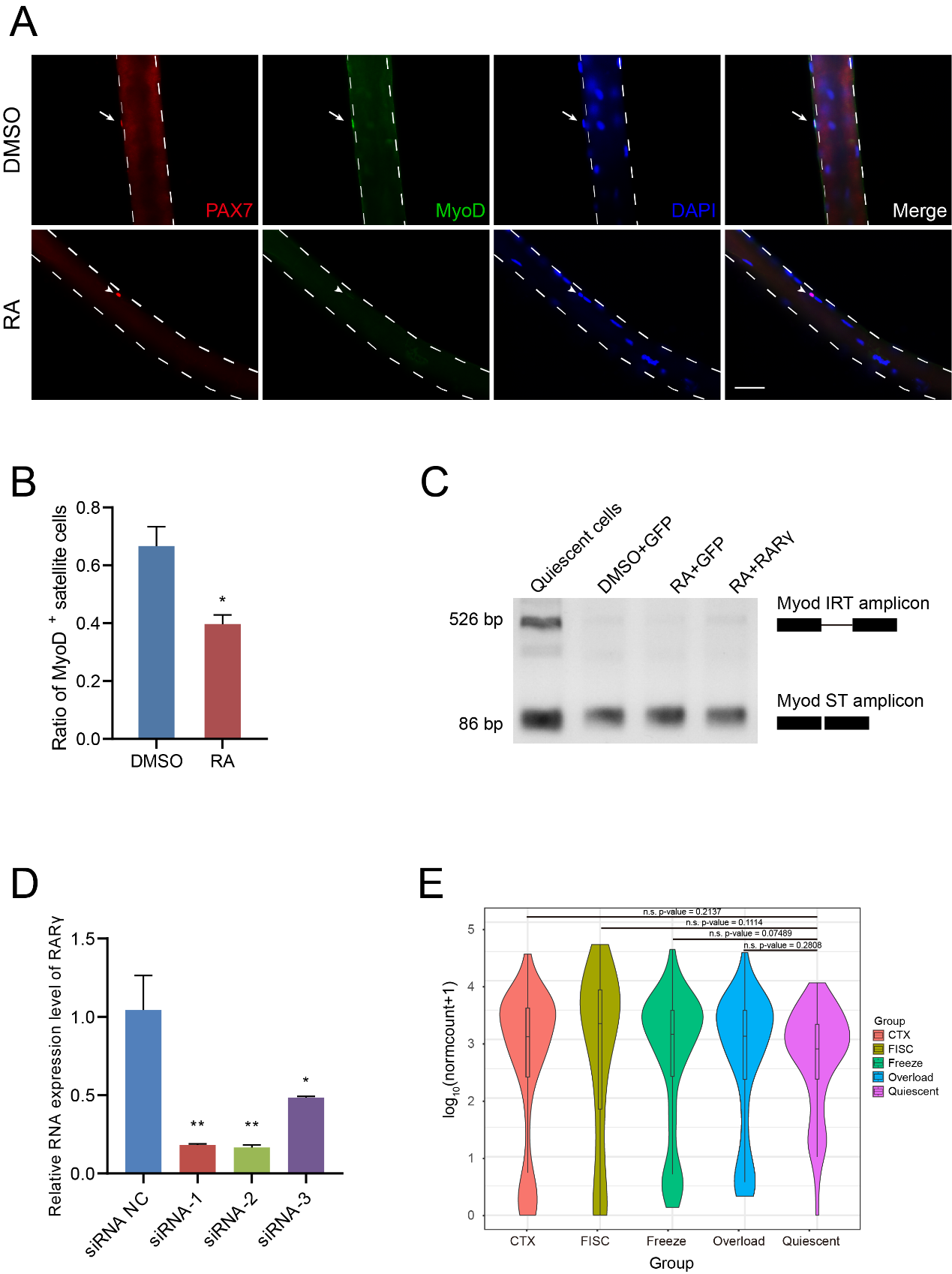


##### Fig. S3: Retinoic acid and RARγ inhibit MyoD protein synthesis, related to Fig. 3.

(A) Fiber-associated MuSCs were treated with DMSO (control) or RA to inhibit MyoD protein expression. Single fibers from extensor digitorum longus (EDL) muscle were incubated with DMSO/RA throughout the isolation and culture process. Fiber-associated MuSCs were stained with PAX7 and MyoD 6 h after EDL isolation. Arrow represents MyoD^+^/PAX7^+^ cell, arrowhead represents MyoD^-^/PAX7^+^ cell.

(B) The proportion of MyoD-positive fiber-associated MuSCs (MyoD^+^/PAX7^+^) were quantified. n = 49 fiber-associated MuSCs.

(C) Semi-quantitative PCR gel electrophoresis analysis of Myod IRTs and STs for quiescent, DMSO+GFP-, RA+GFP- or RA+RARγ-treated MuSCs. IRT amplicon size 526 bp. ST amplicon size 86 bp.

(D) RT–qPCR analysis of RARγ gene in MuSCs treated with 3 independent anti-RARγ siRNA or negative control siRNA. Expression levels were normalized with Gapdh. siRNA-1 was used in the following experiments. n=3 independent assays.

(E) Violin plot of mRNA expression level of eukaryotic translation initiation factor(eIF) related genes for quiescent, CTX-, freeze-, overload- and isolation-induced activated MuSCs.

Scale bar: 50 µm in (A). Statistical results in (D) expressed as the mean ± SEM. n.s., not significant. *p<0.05, **p<0.01.


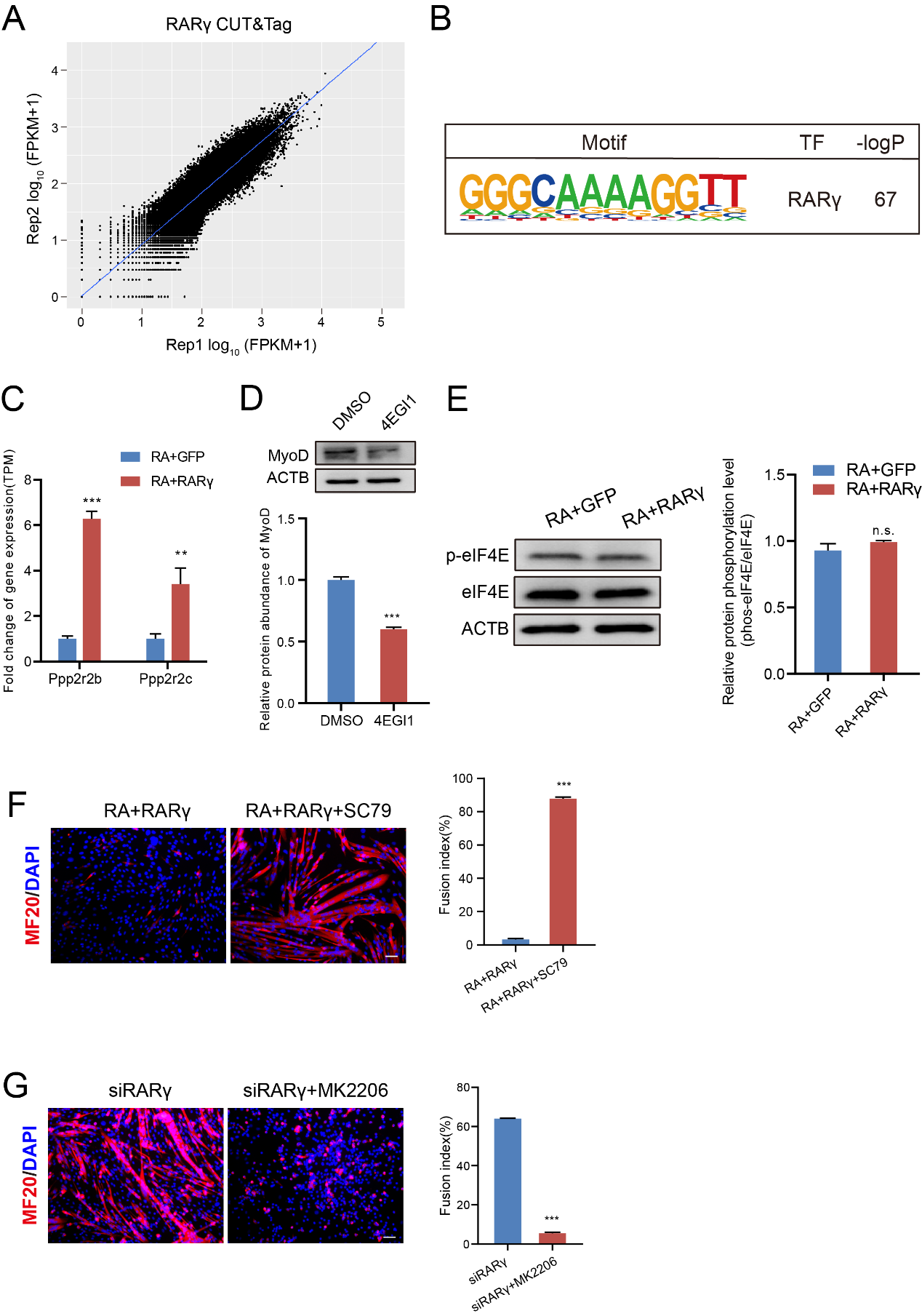


##### Fig. S4: RARγ affects MyoD protein synthesis via the Akt/eIF4EBP1 signaling cascade, related to Fig. 4.

1. Scatter plot showed the repeatability of CUT&Tag data.

(B) Motif analysis represented the genome binding site enrichment of RARγ in CUT&Tag data.

(C) Fold change of Ppp2r2b and Ppp2r2c gene expression (TPM) in RNA-seq data of RA+GFP- or RA+RARγ-treated MuSCs.

(D) Upper: Western blot analysis of MyoD in MuSCs treated with 4EGI1 or DMSO. Bottom: Integrated density of Western blot bands was quantified. n=3 independent assays.

(E) Left: Western blot analysis of total eIF4E and phosphorylated eIF4E (p-eIF4E) in RA+GFP- or RA+RARγ-treated cells. Right: Integrated density of Western blot bands was quantified to determine the ratio of phosphorylated eIF4E. n=3 independent assays.

(F and G) Left: MuSCs treated with (F) RA+RARγ or RA+RARγ+SC79, (G) siRARγ or siRARγ+MK2206 were induced differentiation and stained with MF20. Right: Fusion index was calculated by quantifying the proportion of nuclei inside myotubes (3 or more nuclei) with the respect to the number of total nuclei. n=3 independent assays/condition, >3,000 cell nuclei counted/assay.

Scale bar, 50 µm in (F) and (G). Statistical results are expressed as the mean ± SEM. n.s., not significant, **p<0.01, ***p<0.001.


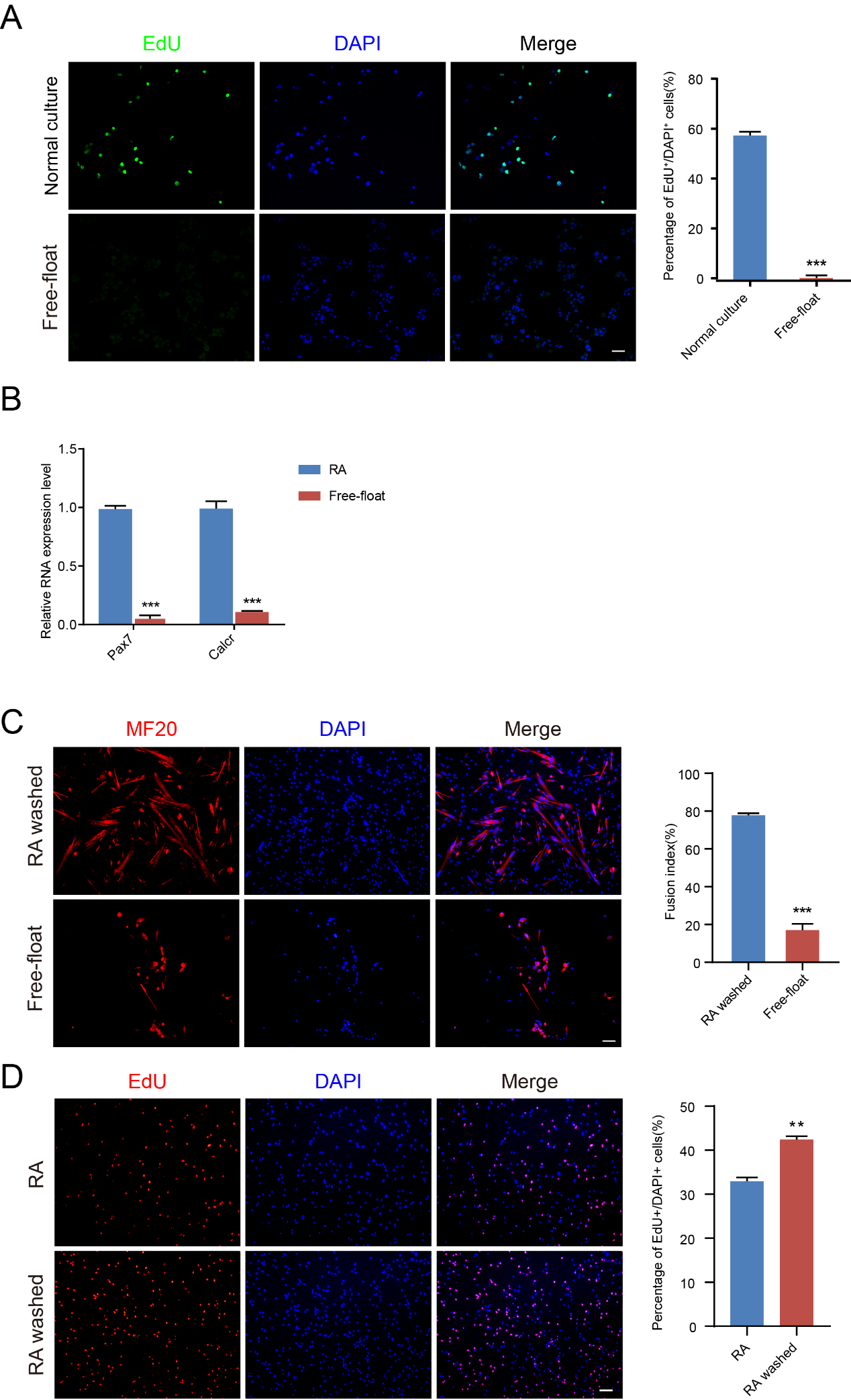


##### Fig. S5: RA signaling maintains the quiescence of satellite cells both in vivo and in vitro, related to Fig. 5.

(A) Left: MuSCs were cultured with 2% methylcellulose-containing medium on bacterial Petri dish for 7 days (free-float) or cultured with normal growth medium on pre-coated dish for 1 day (normal culture) and EdU assay were performed. Right: EdU-positive MuSCs were quantified. n=3 independent assays/condition, >1,000 cells counted/assay.

(B) RT–qPCR analysis of *Pax7* and *Calcr* genes in RA treated and free-float cells. Expression levels were normalized with Gapdh. n=3 independent assays.

(C) Left: RA washed cells and re-adherent free-float cells were induced differentiation and stained with MF20. Right: Fusion index was calculated by quantifying the proportion of nuclei inside myotubes (3 or more nuclei) with the respect to the number of total nuclei. n=3 independent assays/condition, >1,000 cell nuclei counted/assay.

(D) Left: Representative pictures showing EdU assays in MuSCs treated with RA or RA washed. Right: Quantification of EdU-positive MuSCs. n=3 independent assays/condition, >2,000 cells counted/assay.

Scale bar, 50 µm in (A), 100 µm in (C) and (D). Statistical results are expressed as the mean ± SEM. n.s., not significant. **p<0.01, ***p<0.001.
