# Supplementary material for: Retinoic acid and RARγ maintain satellite cell quiescence through regulation of translation initiation
Source: Cell Death Dis. 2022 Sep 29;13(9):838. doi: 10.1038/s41419-022-05284-9 (PMC9522790; doi:10.1038/s41419-022-05284-9)

Figure 3D:

MyoG

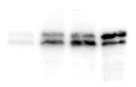

ACTB

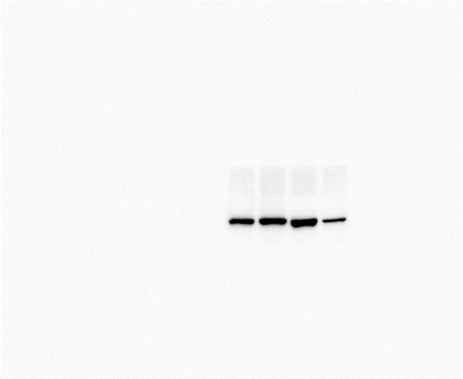

Fiugre 3E:

MyoD

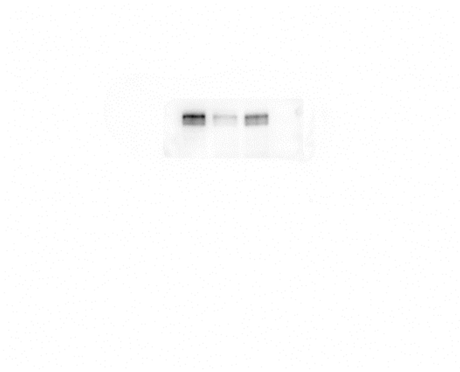

PAX7

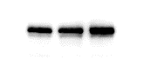

ACTB

Figure 3F:

MyoD

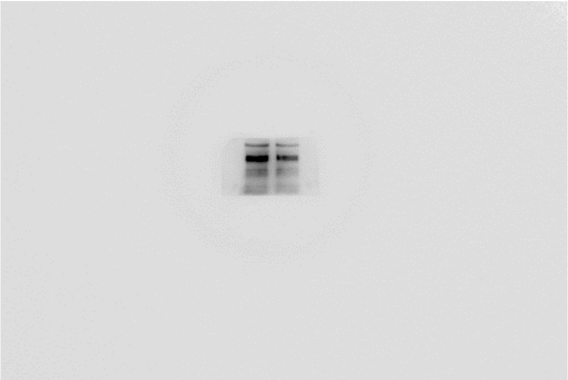

ACTB

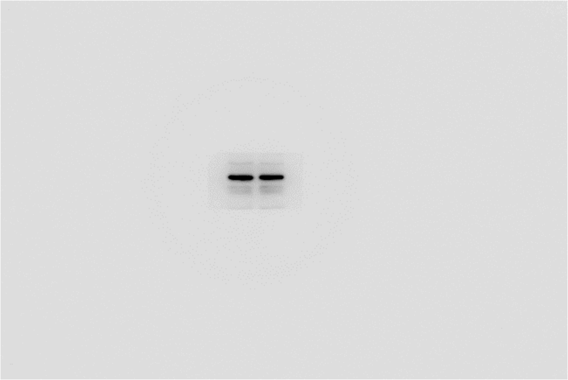

Figure 4F:

P-Akt

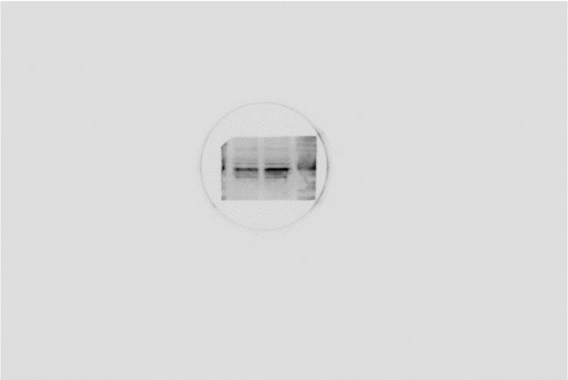

Akt

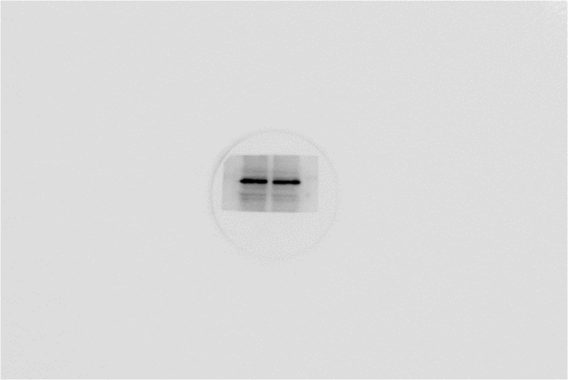

ACTB

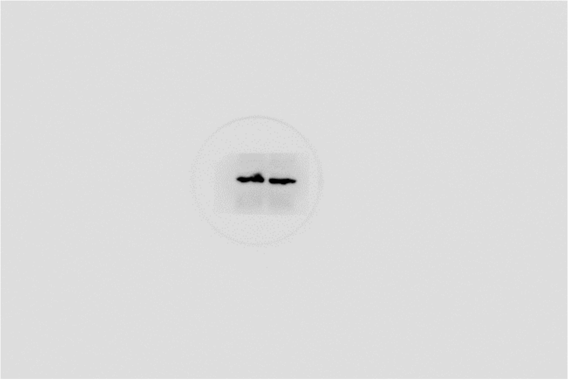

Figure 4G:

p-eIF4EBP1

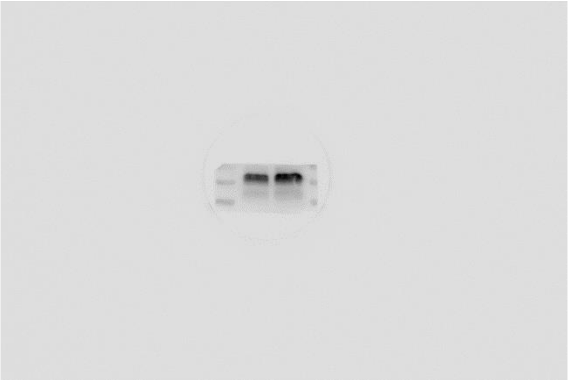

eIF4EBP1

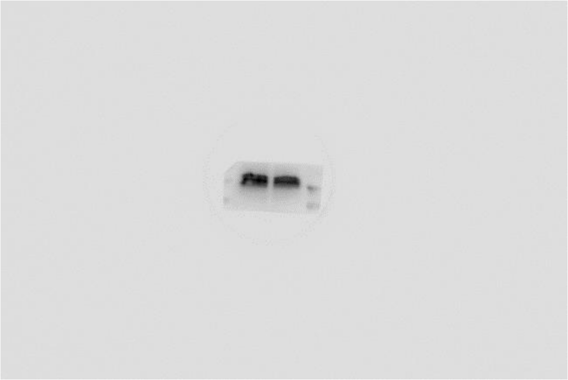

ACTB

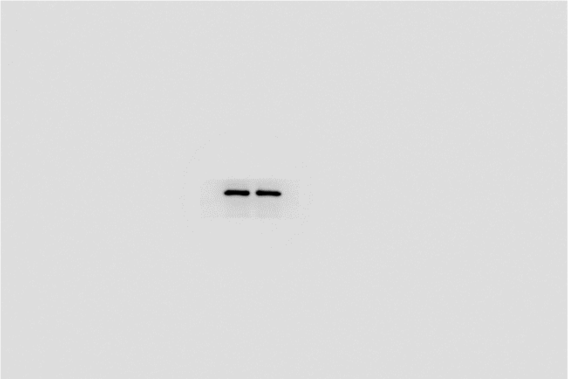

Figure 4H and 4I:

eIF4EBP1

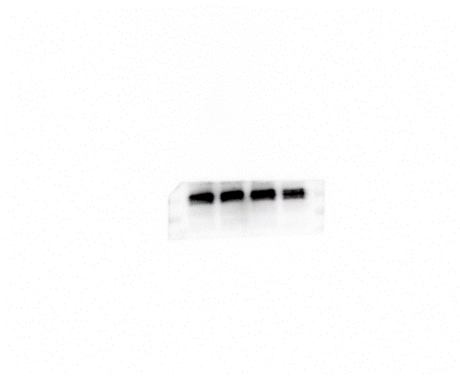

p-eIF4EBP1

Actb

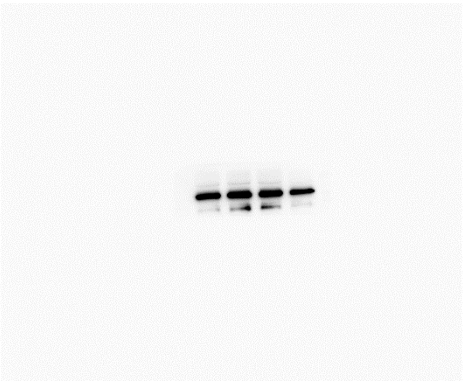

Figure 4H:

MyoD

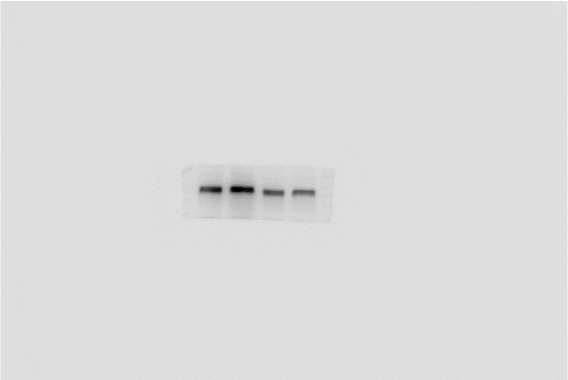

Figure 4I:

MyoD

Figure s4D:

MyoD

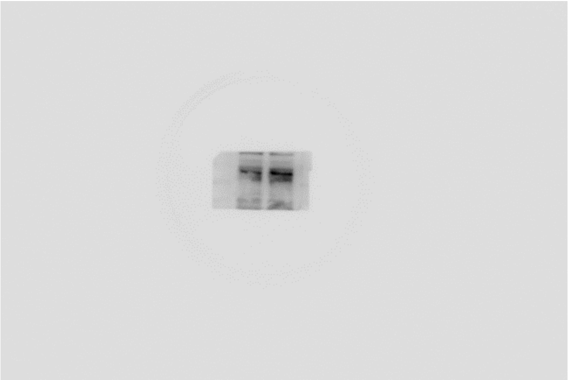

ACTB

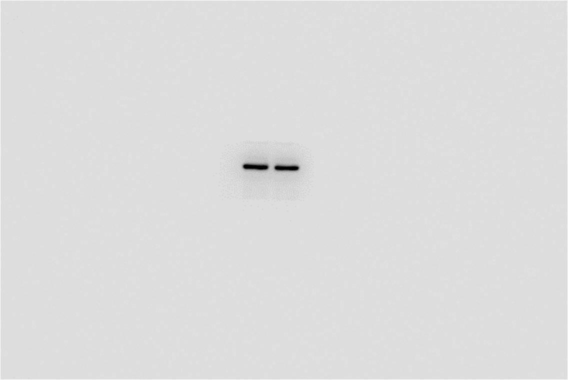

Figure s4E:

p-eIF4E

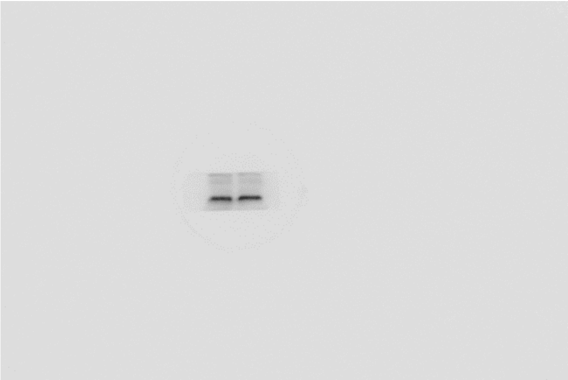

eIF4E

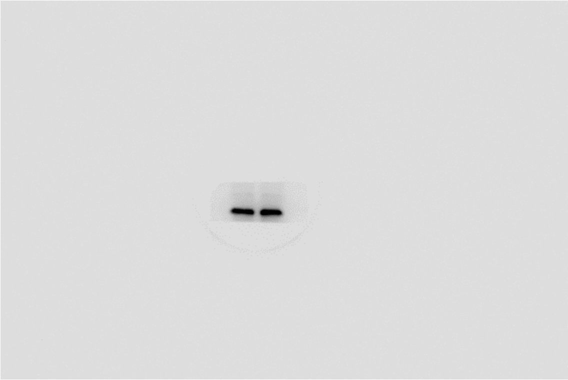

ACTB

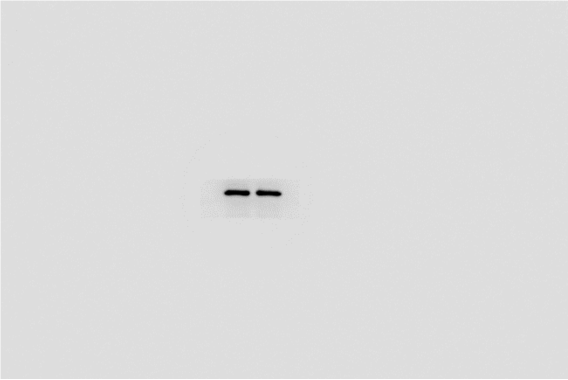

Supplement: Supplementary file 3 — Original western blots [file 41419_2022_5284_MOESM3_ESM.pdf]
