# Supplementary material for: Retinoic acid and RARγ maintain satellite cell quiescence through regulation of translation initiation
Source: Cell Death Dis. 2022 Sep 29;13(9):838. doi: 10.1038/s41419-022-05284-9 (PMC9522790; doi:10.1038/s41419-022-05284-9)
Supplement: Supplementary file 6 — Change of authorship request form [file 41419_2022_5284_MOESM6_ESM.pdf]

SPRINGER NATURE

Change of authorship request form - Journals (pre-acceptance)

Important information. Please read.

- This form should be used by authors to request any change in authorship (adding/deleting authors) including changes in corresponding authors. This form should not be used for name changes. Please fully complete all sections. Use black ink and block capitals and provide each author's full name with the given name first followed by the family name.

- By signing this declaration, all authors guarantee that the order of the authors are in accordance with their scientific contribution, if applicable as different conventions apply per discipline, and that only authors have been added who made a meaningful contribution to the work.

- Please note, in author collaborations where there is formal agreement for representing the collaboration, it is sufficient for the representative or legal guarantor (usually the corresponding author) to complete and sign the Authorship Change Form on behalf of all authors, **next to the added/removed author(s)**. **(Complete Section 3, followed by Section 6.)**
  In author collaborations where there is no formal agreement for representing the collaboration and **there are more than 10 authors**, one may sign for all, provided the signer appends correspondence that attests that each of the authors have agreed to the change **and the added/removed authors sign the form**. **(Complete Section 3, followed by Section 6.)**

- Please note, we cannot investigate or mediate any authorship disputes. If you are unable to obtain agreement from all authors (including those who you wish to be removed) you must refer the matter to your institution(s) for investigation. Please inform us if you need to do this.

- If you are not able to return a fully completed form within **30 days** of the date that it was sent to the author requesting the change, we may have to withdraw your manuscript. We cannot publish manuscripts where authorship has not been agreed by all authors (including those who have been removed).

- Incomplete forms will be rejected.

- Please return/upload this form, fully completed, to the Journals Editorial Office. The Journal and/or Publisher will consider the information you have provided to decide whether to approve the proposed change in authorship. We may decide to contact your institution for more information or undertake a further investigation, if appropriate, before making a final decision.

SPRINGER NATURE

Change of authorship request form - Journals (pre-acceptance)

Section 1: Please provide the current title of manuscript

Manuscript ID no.: CDDIS-22-2061R

Title: Retinoic acid and RARγ maintain satellite cell quiescence through regulation of translation initiation

Section 2: Please provide the previous authorship, in the order shown on the manuscript before the changes were introduced. Please indicate the corresponding author by adding (CA) behind the name.

	First name(s)	Family name	ORCID or SCOPUS id, if available
1st author	Wenzhe	Luo	
2nd author	Yueyuan	Xu	
3rd author	Ruige	Liu	
4th author	Yinlong	Liao	
5th author	Sheng	Wang	
6th author	Haoyuan	Zhang	
7th author	Heng (CA)	Wang	ORCID: 0000-0002-1727-9226
8th author			
9th author			
10th author			

Please use an additional sheet if there are more than 10 authors.

SPRINGER NATURE Springer Nature is one of the world's leading global research, educational and professional publishers, created in May 2015 through the combination of Nature Publishing Group, Palgrave Macmillan, Macmillan Education and Springer Science+Business Media.

SPRINGER NATURE

Change of authorship request form - Journals (pre-acceptance)

Section 3: Please provide a justification for change. Please use this section to explain your reasons for changing the authorship of your manuscript, e.g. what necessitated the change in authorship? Please refer to the (journal) policy pages for more information about authorship. Please explain why omitted authors were not originally included and/or why authors were removed on the submitted manuscript.

After discussion among the authors, it was agreed that the 7th author (Xinyun Li) had made sufficient effort to revise the manuscript, so we decided to add his name to the list of authors. Xinyun Li, together with other authors, approved the final version of manuscript and agreed to be accountable for all aspects of the work in ensuring that questions related to the accuracy or integrity of any part of the work are appropriately investigated and resolved.

Xinyun Li was not originally included because he made substantial contributions to the manuscript revision process. Combined with his earlier efforts in experimental design, so we agreed that his contribution to the overall work was sufficient to be included in the author list.

Section 4: Proposed new authorship. Please provide your new authorship list in the order you would like it to appear on the manuscript. Please indicate the corresponding author by adding (CA) behind the name. <u>If the Corresponding Author has changed, please indicate the reason under section 3</u>.

	First name(s)	Family name (this name will appear in full on the final publication and will be searchable in various abstract and indexing databases)	Affiliated institute	E-mail address
1st author	Wenzhe	Luo	Huazhong Agricultural University; Shandong Agricultural University	wenzhe_luo@webmail.hzau.edu.cn
2nd author	Yueyuan	Xu	Huazhong Agricultural University	yyxu@webmail.hzau.edu.cn
3rd author	Ruige	Liu	Huazhong Agricultural University	lrg202@126.com
4th author	Yinlong	Liao	Huazhong Agricultural University	lylhzau.edu.cn@webmail.hzau.edu.cn
5th author	Sheng	Wang	Huazhong Agricultural University	wangsheng@webmail.hzau.edu.cn
6th author	Haoyuan	Zhang	Huazhong Agricultural University	zhanghaoyuan@webmail.hzau.edu.cn
7th author	Xinyun	Li	Huazhong Agricultural University	xyli@mail.hzau.edu.cn
8th author	Heng (CA)	Wang	Huazhong Agricultural University; Shandong Agricultural University	wangheng@mail.hzau.edu.cn
9th author				
10th author				

Please use an additional sheet if there are more than 10 authors.

SPRINGER NATURE Springer Nature is one of the world's leading global research, educational and professional publishers, created in May 2015 through the combination of Nature Publishing Group, Palgrave Macmillan, Macmillan Education and Springer Science+Business Media.

SPRINGER NATURE

Change of authorship request form - Journals (pre-acceptance)

Section 5: Author contribution, Acknowledgement and Disclosures. Please use this section to provide a new disclosure statement and, if appropriate, acknowledge any contributors who have been removed as authors and ensure you state what contribution any new authors made (if applicable per the journal or book (series) policy). Please ensure these are updated in your manuscript - after approval of the change(s) - as our production department will not transfer the information in this form to your manuscript.

New acknowledgements:

This work was supported by National Key R&D Program of China (2021YFF1000601), National Natural Science Foundation of China (31972536, 32101517), Natural Science Foundation of Hubei Province (2020CFA006), Key Research and Development Program of Guangxi (AB19245030).

New Disclosures (financial and non-financial interests, funding):

The authors declare no competing interests.

New Author Contributions statement (if applicable per the journal policy):

WL conducted most of the experiments and wrote the paper; SW and YX analyzed the RNA-seq and CUT&TAG high-throughput sequencing data; RL and HZ manipulated mice; YL performed the CUT&TAG experiment; XL and HW designed the experiments and revised the paper. All authors read and approved the final manuscript.

State 'Not applicable' if there are no new authors.

SPRINGER NATURE Springer Nature is one of the world's leading global research, educational and professional publishers, created in May 2015 through the combination of Nature Publishing Group, Palgrave Macmillan, Macmillan Education and Springer Science+Business Media.

Change of authorship request form - Journals (pre-acceptance)

Section 6: Declaration of agreement. *All* authors, unchanged, new and removed *must* sign this declaration.
(NB: Please print the form, (docu)-sign and return/upload a scanned copy. Please note that signatures that have been inserted as an image file are acceptable as long as it is handwritten. Typed names in the signature box are unacceptable.) *** Please delete as appropriate. Delete all of the bold if you were on the original authorship list and are remaining as an author.**

	First name	Family name		Signature	Date
1st author	Wenzhe	Luo	I agree to the proposed new authorship shown in section 4 and the proposed change in corresponding author	WENZHE LUO	2022.9.15
2nd author	Yueyuan	Xu	I agree to the proposed new authorship shown in section 4 and the proposed change in corresponding author	YUEYUAN XU	2022.9.15
3rd author	Ruige	Liu	I agree to the proposed new authorship shown in section 4 and the proposed change in corresponding author	RUIGE LIU	2022.9.15
4th authors	Yinlong	Liao	I agree to the proposed new authorship shown in section 4 and the proposed change in corresponding author	YINLONG LIAO	2022.9.15
5th author	Sheng	Wang	I agree to the proposed new authorship shown in section 4 and the proposed change in corresponding author	SHĔNG WANG	2022.9.15
6th author	Haoyuan	Zhang	I agree to the proposed new authorship shown in section 4 and the proposed change in corresponding author	HAOYUAN ZHANG	2022.9.15
7th author	Xinyun	Li	I agree to the proposed new authorship shown in section 4 and the addition of my name to the authorship list and the proposed change in corresponding author	XINYUN LI	2022.9.15

SPRINGER NATURE Springer Nature is one of the world's leading global research, educational and professional publishers, created in May 2015 through the combination of Nature Publishing Group, Palgrave Macmillan, Macmillan Education and Springer Science+Business Media.

Change of authorship request form - Journals (pre-acceptance)

	First name	Family name		Signature	Date
8th author	Heng	Wang	I agree to the proposed new authorship shown in section 4 and the proposed change in corresponding author	HENG WANG	2022.9.15
9th author			I agree to the proposed new authorship shown in section 4 **/and the addition/removal*of my name to the authorship list** /and the proposed change in corresponding author		
10th author			I agree to the proposed new authorship shown in section 4 **/and the addition/removal*of my name to the authorship list** /and the proposed change in corresponding author		

Please use an additional sheet if there are more than 10 authors.

In case of author collaborations with formal agreement:

	Name of consortium/consortia	First name	Family name		Signature	Date
Representative/legal guarantor				I agree to the proposed new authorship shown in section 4 **/and the addition/removal*of my name to the authorship list** /and the proposed change in corresponding author		

Both added/removed authors should complete the information in the first table under Section 6.

---- End of form ----

SPRINGER NATURE Springer Nature is one of the world's leading global research, educational and professional publishers, created in May 2015 through the combination of Nature Publishing Group, Palgrave Macmillan, Macmillan Education and Springer Science+Business Media.
